# Rice glycosyltransferase OsDUGT1 is involved in heat stress tolerance by glycosylating flavonoids and regulating flavonoid metabolism

**DOI:** 10.3389/fpls.2024.1516990

**Published:** 2025-01-13

**Authors:** Guang-rui Dong, Shu-man Zhao, Yi Ding, Yu-qing Ma, Xing-mei Ma, Chong-lin Liu, Bing-kai Hou

**Affiliations:** The Key Laboratory of Plant Development and Environmental Adaptation Biology, Ministry of Education, Shandong Key Laboratory of Precision Molecular Crop Design and Breeding, School of Life Sciences, Shandong University, Qingdao, China

**Keywords:** rice, *Arabidopsis thaliana*, glycosyltransferase, OsDUGT1, flavonoids, heat stress tolerance

## Abstract

One significant environmental element influencing the growth and yield of rice (*Oryza sativa* L.) is high temperature. Nevertheless, the mechanism by which rice responds to high temperature is not fully understood. A rice glycosyltransferase gene, *OsDUGT1*, was identified as a heat-responsive gene in this investigation. Its function was studied by overexpression and knockout methods. The results showed that under heat stress, *OsDUGT1* overexpression lines (*OsDUGT1*-OE) increased the survival rate of rice, while *Osdugt1* knockout lines (*Osdugt1*-ko) decreased the survival rate compared to wild type (ZH11). In addition to rice, heat stress tolerance was also improved by ectopic expression of *OsDUGT1* in transgenic *Arabidopsis thaliana* plants. We observed that ROS scavenging ability, malondialdehyde accumulation, and the ion leakage are relevant to the expression level of *OsDUGT1*. Through enzyme activity analysis, we found that *OsDUGT1* could glycosylate flavonoid compounds. Correspondingly, the loss of *OsDUGT1* function caused a significant decrease in endogenous flavonoid accumulation in rice, which was demonstrated by our metabolomics analysis. Additionally, our transcriptomic analysis of *Osdugt1* mutant lines under heat stress condition indicated that mutation of *OsDUGT1* can reduce the transcriptional activity of heat response related genes, antioxidant enzyme genes and other genes involved in the flavonoid biosynthetic pathway. In summary, our work revealed that *OsDUGT1* plays a crucial role in adjusting and balancing the overall plant metabolism and transcription under heat stress through glycosylation of flavonoids, and offers a key prospect gene for breeding efforts to enhance crop heat tolerance under the trend of climate warming all over the globe.

## Highlights

- The rice glycosyltransferase OsDUGT1 has been shown to be a heatresponsive gene. It plays an important role in improving heat tolerance by glycosylating flavonoids and regulating flavonoid metabolism.

## Introduction

Heat stress can adversely affect plant growth and development, physiological processes and yield (Gupta et al., 2015; Sharma et al., 2019; Hassan et al., 2020; Guihur et al., 2022). For instance, the yield of rice (*Oryza sativa* L.) is estimated to decrease by 3.2% for every 1°C increase in the average global temperature (Zhao et al., 2017). According to recent literature, 10% yield reduction will occur for every 1°C increase of night-time temperature during the rice reproductive stage. In the case of *Japonica* rice in particular, field temperatures of more than 35°C tend to cause significant yield losses(Chang et al., 2024). Excessive formation of reactive oxygen species (ROS) is a key effect of heat stress, which results in oxidative stress (Das et al., 2024). ROS are considered the byproducts of aerobic metabolism. The multifunctional cellular redox sensing systems and ROS-dependent regulatory networks have the capacity to influence the majority of cellular processes, from transcription to translation and metabolism, and serve as integral early signaling components in the appropriate responses to environmental stresses (Denjalli et al., 2024). In plants, the main forms of ROS are superoxide anion (O^−2^), singlet oxygen (1O^2^), hydroxyl radical (HO·) and hydrogen peroxide (H_2_O_2_) (Bheri and Pandey, 2018). When the antioxidant capacity of cells is exceeded by the production of reactive oxygen species, cellular macromolecules (e.g., lipids, proteins, and DNA) are susceptible to damage. Under normal circumstances, ROS generation and clearance are balanced. Nevertheless, under conditions of heat stress, this equilibrium is disturbed, resulting in an elevated production of ROS (Zhao et al., 2018a). One of the primary mechanisms by which plants remove ROS in response to stress is the activation of the antioxidant enzyme system, including ascorbate peroxidase (APX), catalase (CAT), superoxide dismutase (SOD), peroxidase (POD), glutathione reductase (GR) and other antioxidant enzymes. Moreover, there was a positive correlation between the activity of antioxidant enzymes and rising temperatures (Zhao et al., 2018b).

When plants are exposed to elevated temperatures, metabolic processes are affected, resulting in protein aggregation, misfolding and denaturation (Jacob et al., 2017). As molecular chaperones, heat shock proteins (HSP) are integral to the sustenance of cellular homeostasis and protein functions. According to the different molecular weight, HSPs can be classified into numerous categories, such as HSP20, HSP60, HSP70, HSP90, HSP100 and so on (Swindell et al., 2007; Dahuja et al., 2022). The promoter region of heat shock proteins contains several conserved heat shock elements, and their expression is mainly under the regulation of heat shock transcription factors (HSFs), which trigger the transcription of *HSP* genes in plants (Kang et al., 2022). For example, in *Arabidopsis thaliana*, HSFA2 plays a major role in resistance to heat stress (Nishizawa et al., 2006; Chan-Schaminet et al., 2009). Overexpressing *OsHsfA2e* and *OsHSFA2dI* can improve rice tolerance to environmental heat resistance (Yokotani et al., 2008; Cheng et al., 2015). *TaHsfA2-10*, TaHsfA2-7 actively regulates heat stress in wheat (Guo et al., 2020; Ma et al., 2023a). These results suggest that HSPs and HSFs are essential for the management of heat stress in plants.

Additionally, secondary metabolism is crucial to plant life since it is a key component of how plants interact with their surroundings (Yang et al., 2005). Plants produce many secondary metabolites during metabolism, such as flavonoids, terpenoids, alkaloids, and phenolic acids. These substances play an important role in grow and development, biotic and abiotic stress tolerance of plants (Yang et al., 2018). Flavonoids are very abundant secondary metabolites in plants and have many biological functions (Treutter, 2006; Zhang Y. M. et al., 2021; Feng et al., 2023). Flavonoids can be used as effective ROS scavengers, helping to remove excess hydroxyl free radicals in plants, thus reducing damage to plant growth and development (Muhlemann et al., 2018; Shen et al., 2022). For example, Postiglione and colleagues found that excessive accumulation of flavonoids in tomato exposed to high temperature could reduce ROS levels, protect pollens from high temperature stress, and thus improve pollen viability, germination rate and tube growth (Postiglione et al., 2024). UDP-dependent glycosyltransferases (UGTs) are members of the glycosyltransferase superfamily, and they usually catalyze the transfer of sugars to secondary metabolites for the formation of a glycosidic bond (Mackenzie et al., 1997). UGTs have been implicated as key players in the response to environmental stress. For example, *AtUGT76E11* in *Arabidopsis thaliana* has been shown to regulate plant flavonoid metabolism and to enhance the plant’s ability to adapt to environmental stress (Li et al., 2017). *GSA1*, encoding UGT83A1 in rice, is involved in redirecting metabolic flux from lignin to flavonoid biosynthetic pathways, and enhancing rice tolerance to abiotic stresses (Dong et al., 2020). UGTs are the largest glycosyltransferase family in plant kingdom and undergo significant expansion during the evolution of plants. However, for most *UGT* genes, their physiological significance in adaptation of plants to live on the land is largely unknown.

In our preliminary screening for stress-response rice *UGT* genes, the evidence that *OsDUGT1* was upregulated under thermal stress condition makes *OsDUGT1* an interesting candidate for our further study. We created transgenic rice plants, including *OsDUGT1* overexpression plants and *Osdugt1* mutant plants, and also created transgenic *Arabidopsis thaliana* plants for the purpose of this study. Our results showed that *OsDUGT1* gene is heat stress-induced and critical for plant heat tolerance response. In addition, we found that OsDUGT1 can catalyze the glycosylation of flavonoids and influence the flavonoids metabolism. Moreover, our metabolomic and transcriptomic data revealed that OsDUGT1 contributes to the global metabolic and transcriptional adjustments of plants upon high temperature challenge. This work provides a theoretical foundation for the mechanisms of heat tolerance in plants, and also potentially promotes the heat-resistant breeding of crop plants under the trend of climate warming all over the globe.

## Materials and methods

### Plant materials and growing conditions

Rice (*Oryza sativa* ssp. japonica) “Zhonghua 11” (ZH11) and *Arabidopsis thaliana* (Ecotype Col-0) served as the study’s wild type plants. In a greenhouse with a 14 h/10 h light-dark cycle, 28°C, and 50%–60% humidity, rice seedlings were cultivated with 1/2 Hoagland’s solution (HS) and stroma (nutritive soil: vermiculite = 3:1), respectively. *Arabidopsis thaliana* seedlings were cultivated with MS medium and stroma (nutritive soil: vermiculite = 3:1) in a controlled environment with a 16 h/8 h light-dark cycle, 100 µmol/s^-1^/m^-2^ of light intensity, 22°C, and 40% - 60% humidity.

### Plasmid construction and genetic transformation

The Hangzhou Biogle Vector Construction Kit (http://www.biogle.cn) was used to generate the CRISPR/Cas9-mediated mutation construct of *OsDUGT1*. To construct the rice overexpression plasmid, the full-length CDS of *OsDUGT1* (LOC_Os01g41430) was amplified from rice cDNA using ApexHF HS DNA Polymerase CL (AG Company, China), and then cloned into the pUN1301 vector under the control of the maize ubiquitin promoter. The *Agrobacterium*-mediated genetic transformation of rice was completed by BioRun Biotechnology Co., Ltd (Wuhan, China). In addition, the full-length cDNA of *OsDUGT1* were cloned into a pBI121 binary vector under the control of *CaMV 35S* promoter, which was subsequently introduced into *Arabidopsis* via the flower-dipping technique in order to achieve ectopic expression of *OsDUGT1*. The primers used to construct the plasmids are listed in **Supplementary Table S1**.

### High temperature stress assays

The high temperature stress experiments of rice and *Arabidopsis* were carried out in the same incubator and same conditions to keep environmental consistency (including temperature, light intensity, light-dark cycle, humidity etc.)

(1) High temperature induction of gene expression: two-week-old rice seedlings were transferred to 45°C high temperature treatment and the samples were collected at 0h, 3h, 6h, 12h, 24h and 48h during treatment. The gene expression levels of *OsDUGT1* at each time point relative to 0h were analyzed using qRT-PCR.
(2) Heat stress treatment of hydroponically grown rice seedlings: All rice seedlings were normally grown in the chamber at 28°C for two weeks. *Osdugt1* knockout lines (ko18, ko75), wild type (ZH11) and *OsDUGT1* overexpressing lines (OE19, OE21) were treated under 45°C for 24h and then recovery for 7 days. The survival rates after recovery were calculated.
(3)Treatment of soil-grown rice seedlings under heat stress: All plants were grown in the soil for about 6 weeks, and then subjected to 45°C high temperature stress treatment (12 h for ko mutant lines, 16 h for OE lines), and then restored in a 28°C greenhouse for 10 days. The survival rate was recorded.
(4) *Arabidopsis* hypocotyl growth assays: *Arabidopsis* seeds were cultivated on the MS medium for three days, then put into a 45°C incubator under high temperature stress for 2~3 h, followed by recovery culture in a 22°C for 3 days in darkness. Photographs were taken to record the hypocotyl growth.
(5) *Arabidopsis* root growth assays: All seeds were grown on MS medium. When the roots grew to about 1cm, transferred to the new 1/2 MS medium, then heat-treated at 45°C for 1 h and followed by recovery culture for 7 days. The phenotype of the plant root growth was photographed.
(6) Heat treatment of media-grown *Arabidopsis* seedlings: All seeds were cultivated on the MS medium for 7 days. Then transfer the seedlings to the new 1/2MS medium and subjected to heat-treatment at 45°C for 2 h, followed by recovery culture at room temperature for 3 days. The phenotype of the plant growth was photographed and survival rate was measured.
(7) High temperature stress of soil-grown *Arabidopsis* seedlings: The seedlings grown on MS medium for 1 week were transplanted into the soil for another week under normal temperature, and then transferred to a 45°C incubator for heat stress treatment of 24 h. After a recovery growth at normal temperature of 22°C for 7 days, the growth phenotype was photographed and survival rate was measured.

### Nitrobluetetrazolium, diaminobenzidine staining and chlorophyll fluorescence measurement

DAB and NBT staining are performed in accordance with the procedure outlined by literature (Dolui et al., 2024), with slight modifications. Rice leaves were collected following a 24 h of heat stress treatment and submerged in 0.5 mg/mL NBT solution in 10 mM phosphate buffer (pH7.6) before being vacuum-infiltrated for ten minutes. Samples were then dyed for 12 h at room temperature in the absence of light. After discarding the dyeing solution, samples were cooked for 10 minutes in the fixed solution (ethanol: lactic acid: glycerin = 3:1:1) until all of the chlorophyll was gone, at which point they were photographed.

Rice leaves with same heat treatment were gathered and submerged in a 1 mg/mL dimethylbenzidine (DAB) solution in 50 mM Tris-HCl (pH3.8) for 15 minutes before being vacuum-infiltrated. Samples were then dyed for 16 h in the dark at room temperature. Following a 15 minutes of decolonization in a water bath at 70°C with 90% ethanol, the pigmented samples were photographed.

Chlorophyll fluorescence parameters were recorded by a PAM fluorometer (Walz; PAM 2500) after dark adaptation (30 min) (Ghorbani et al., 2021).

### RNA extraction and qRT-PCR analysis

The Plant RNA Kit (Vazyme Biotech Co., Nanjing, China) was used to extract total RNA from *Arabidopsis* and rice seedlings. The PrimeScript RT reagent kit with gDNA Eraser (Perfect Real Time) (Accurate Biology, Hunan, China) was used for reverse transcription and cDNA synthesis. The SYBR Green Supermix (Transgen, Beijing, China) was used for qRT-PCR. Pre-denaturation was done at 95°C for 3 minutes, and then there were 45 cycles (5 s at 95°C, 30 s at 60°C, and 30 s at 72°C). Data was taken at 72°C for each cycle. *OsACTIN1* and *OsUBIQ1* were used as the internal reference genes for rice gene analysis. *AtACTIN2* and *AtTUB2* were used as the internal reference genes for *Arabidopsis* gene analysis. Gene expression levels were normalized to these reference genes and the fold change in the expression of each target gene was calculated by using the 2^−ΔΔCt^ method. **Supplementary Table S1** contained the primers for the qRT-PCR study.

### Measurement of H_2_O_2_, CAT, SOD, POD, MDA and ion leakage

Two-weeks-old *OsDUGT1* transgenic and wild type rice seedlings were heat-treated for 24 h at 45°C, whereas untreated seedlings served as the control. According to the manufacturer’s instructions (Shanghai Sangon Biotech, Shanghai, China), the hydrogen peroxide (H_2_O_2_) content, catalase (CAT, EC 1.11.1.6), superoxide dismutase (SOD, EC 1.15.1.1), peroxidase (POD, EC 1.11.1.7), and malondialdehyde (MDA) contents were measured using the H_2_O_2_ Assay Kit (D799774-0100), CAT Test Kit (D799598-0100), SOD Assay Kit (D799594-0100), POD Assay Kit (D799592-0100), and MDA Assay Kit (D799762-0100), respectively. As previously mentioned, the relative ion leakage rate was measured (Fang et al., 2015).

### *Escherichia coli* protein expression and enzyme activity test

The pGEX-4T vector (Takara, Japan) was used to clone the whole *OsDUGT1* cDNA sequence. The *E.coli* strain BL21 was inoculated with the constructs, and the transformed single colony was cultivated for approximately 12 h at 37°C in LB medium containing 100 mg/mL ampicillin. The culture solution was then transferred to 75 mL 2×YT media containing 50 mg/mL ampicillin at 37°C until the OD_600_ reached 0.6~0.9. After adding 1 mM isopropyl β-D-1-thiogalactopyranoside (IPTG) and incubating for 24 h at 16°C, the OsDUGT1 fusion protein was expressed. Centrifugation at 5000 × rpm for 5 minutes at 4°C was used to harvest the cells, and they were then re-suspended in 25 mL of PBS solution containing 50 µL of phenylmethylsulfonyl fluoride (PMSF, 100 mM). After being lysed using an ultrasonic cell crusher, the supernatant was got by centrifuging suspension at 7000 × rpm for 25 minutes at 4°C. 100 µL of GST agarose beads were added and the tube was incubated for one hour at room temperature. The purified protein was then eluted using elution buffer.

A 100 µL aliquot of reaction mixture including 0.1 mM substrate, 2.5 mM MgSO4, 10 mM KCl, 100 mM Tris-HCl (pH8.0), 14 mM 2-mercaptoethanol, 5 mM UDP-glucose, and 1 μg of OsDUGT1 fusion protein was used for the enzyme activity experiment. After 30 minutes of incubation at 30°C, 100 µL of methanol was added to the reaction mixtures to quench the reaction. The mixtures of enzymatic reactions were centrifuged at 4°C for 10 minutes at 12,000 × rpm. Liquid chromatography tandem mass spectrometry (LC-MS/MS) analysis was subsequently performed on the supernatant.

### Secondary metabolome analysis

The secondary metabolomics experiment was carried out by Metware Co. (Wuhan, China). First, the sample was freeze-dried with Scientz-100F, and then powdered with MM 400, Retsch (30 Hz, 1.5 min). Next, 50 mg of the sample was weighed and added to pre-cooled 70% methanol for extraction. Following centrifugation (rotation speed 12000 rpm, 3 minutes), the sample was filtered using a microporous filter membrane (0.22 μm pore size), the supernatant was absorbed for UPLC-MS/MS analysis. The following are the primary liquid phase conditions: 1 Column: Agilent SB-C18 1.8 µm, 2.1 mm, 100 mm; (2) The mobile phase consists of acetonitrile (with 0.1% formic acid added) and ultra-pure water (phase A); (3) elution gradient: A/B phase: 95:5 V/V at 0 min, 5:95 V/V at 9.0 min, 5:95 V/V at 10.0 min, 95:5 V/V at 11.1 min, 95:5 V/V at 14.0 min; (4) flow rate: 0.35mL/min;temperature:40°C; injection volume: 2 µL. Next, we attached an ESI-triple quadrupole-linear ion trap (QTRAP)-MS to the effluent. Triple quadrupole (QQQ) scanning and liquid chromatographic analysis were performed using the Q TRAP mass spectrometer and API 6500 Q TRAP LC/MS/MS system. This system ran in positive ion mode and had an ESI Turbo Ion-Spray interface. The electrospray ionization (ESI) temperature of 550°C and the ion spray voltage (IS) of 5500 V (positive ion mode) or -4500 V (negative ion mode) are the primary mass spectrum conditions. The collision-induced ionization parameters are set to high, and the ion source gas I, gas II, and curtain gas were set to 50, 60, and 25 psi, respectively. QQQ scans obtained with nitrogen as collision gas for MRM experiments. Triple quadrupole mass spectrometry with multiple reaction monitoring (MRM) was used to quantify metabolites. The program Analyst 1.6.3 was used to process the mass spectrum data.

### Preparing RNA-seq libraries and analyzing data

Using the Trizol Reagent, total RNA was extracted from rice seedlings. Illumina NovaSeq (Wuhan, China) was used to implement the RNA-seq libraries. The fastqc software (version: 0.11.5) was used to analyze the raw data. The hisat2 software (version: 2.0.1-beta) was used to compare the clean read data to the reference genome (https://rapdb.dna.affrc.go.jp). All RNA-seq libraries’ quantitative expression analyses were compiled using FeatureCounts (version: v1.6.0). Edge R package was used to evaluate the differentially expressed genes (DEGs) compared to wild type. Log2 FC ≥1 and p value ≤ 0.05 were regarded as significant differential genes. GO and KEGG enrichment analyses were performed on these DEGs.

### Analysis of statistics

At least three biological replicates and three technical replicates each provided the data present in this study. The mean ± SD value was used to report the results. One-way ANOVA followed by Tukey’s test was performed using SPSS software.

## Results

### *OsDUGT1* is a high temperature inducible gene in rice

**Figure 1:**
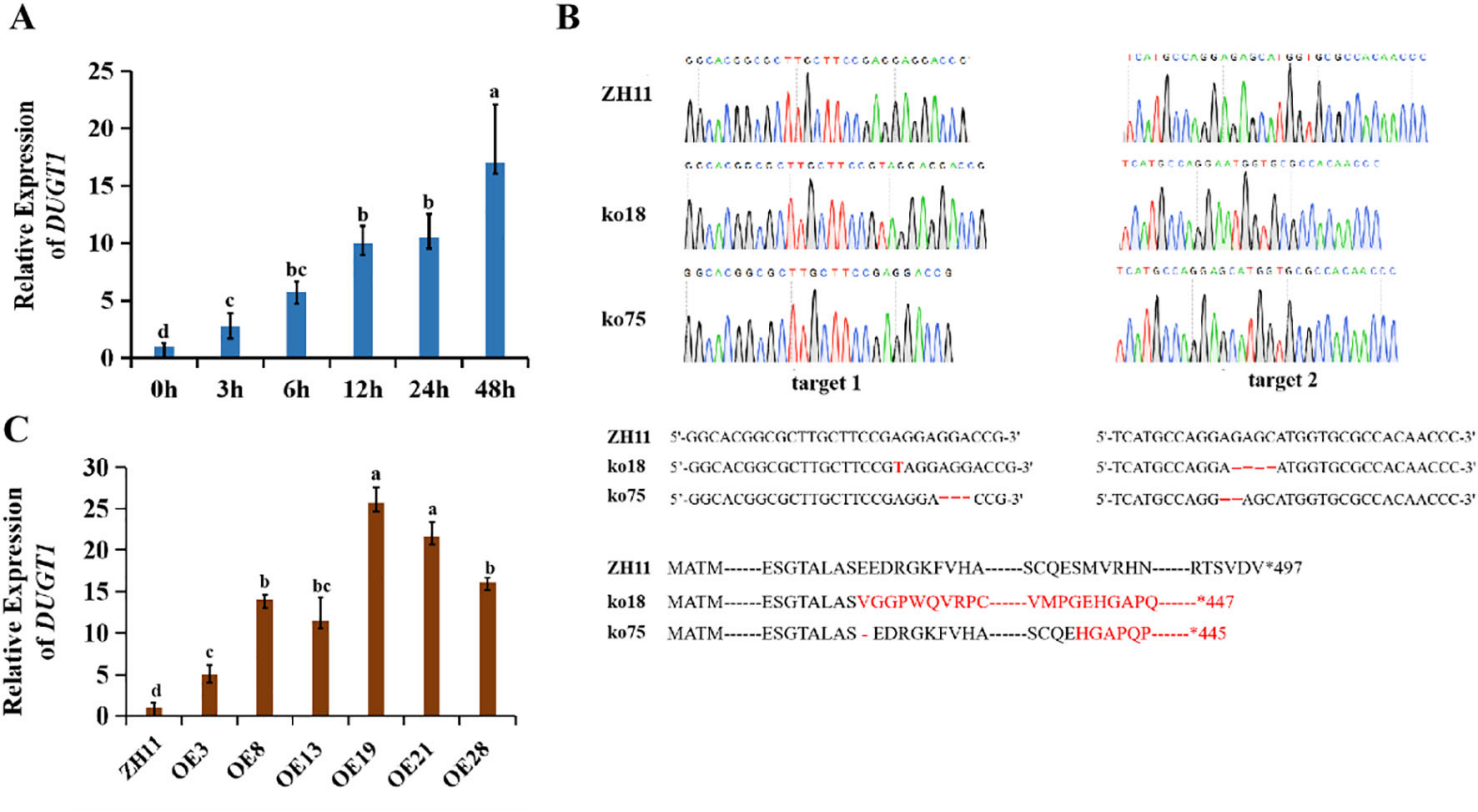
The induced expression of Os*DUGT1* and the preparation of Os*DUGT1* transgenic rice plants. **(A)** Os*DUGT1* expression patterns of rice under 45°C treatment through the qRT-PCR analysis. **(B)** Preparation of *Osdugt1* mutants: ko18 harbors 1-bp insertion at target site 1 and 4-bp deletion at target site 2. ko75 harbors 3-bp deletion at target 1 and 2-bp deletion at target 2. The amino acid sequence in black color indicates the wild type amino acids and the red indicates those from frameshift. **(C)** qRT-PCR analysis showing the expression levels of *OsDUGT1*-overexpressing lines of rice. ZH11 was the wild type rice used in this study. The average number with the same letter (a, b, c, d) in Tukey's test indicates no significant difference at P < 0.05.

Using Genevestigator data platform (https://genevestigator.com/gv), we searched the *UGT* genes in rice genome to find some members that were up-regulated or down-regulated by high temperature. It was noticed that *OsDUGT1* gene (LOC_Os01g41430.1) was annotated as high temperature inducible in this database. To confirm this, we treated wild type rice (ZH11) with high temperature and detected *OsDUGT1* expression during treatment by qRT-PCR. We found that *OsDUGT1* transcription was significantly increased under heat stress (**Figure 1A**; **Supplementary Figure S1A**), suggesting it is high temperature inducible gene. To further explore its function and mechanism, we created its knockout mutants using CRISPR/Cas9 strategy, and obtained its homozygous mutant lines *Osdugt1-*ko18 *and Osdugt1-*ko75 (**Figure 1B**). Besides, we also created and identified several overexpression lines of *OsDUGT1* (OE19, OE21, etc.) (**Figure 1C**; **Supplementary Figure S1B**). These lines were used for subsequent studies.

### *OsDUGT1* confers heat tolerance in rice

**Figure 2:**
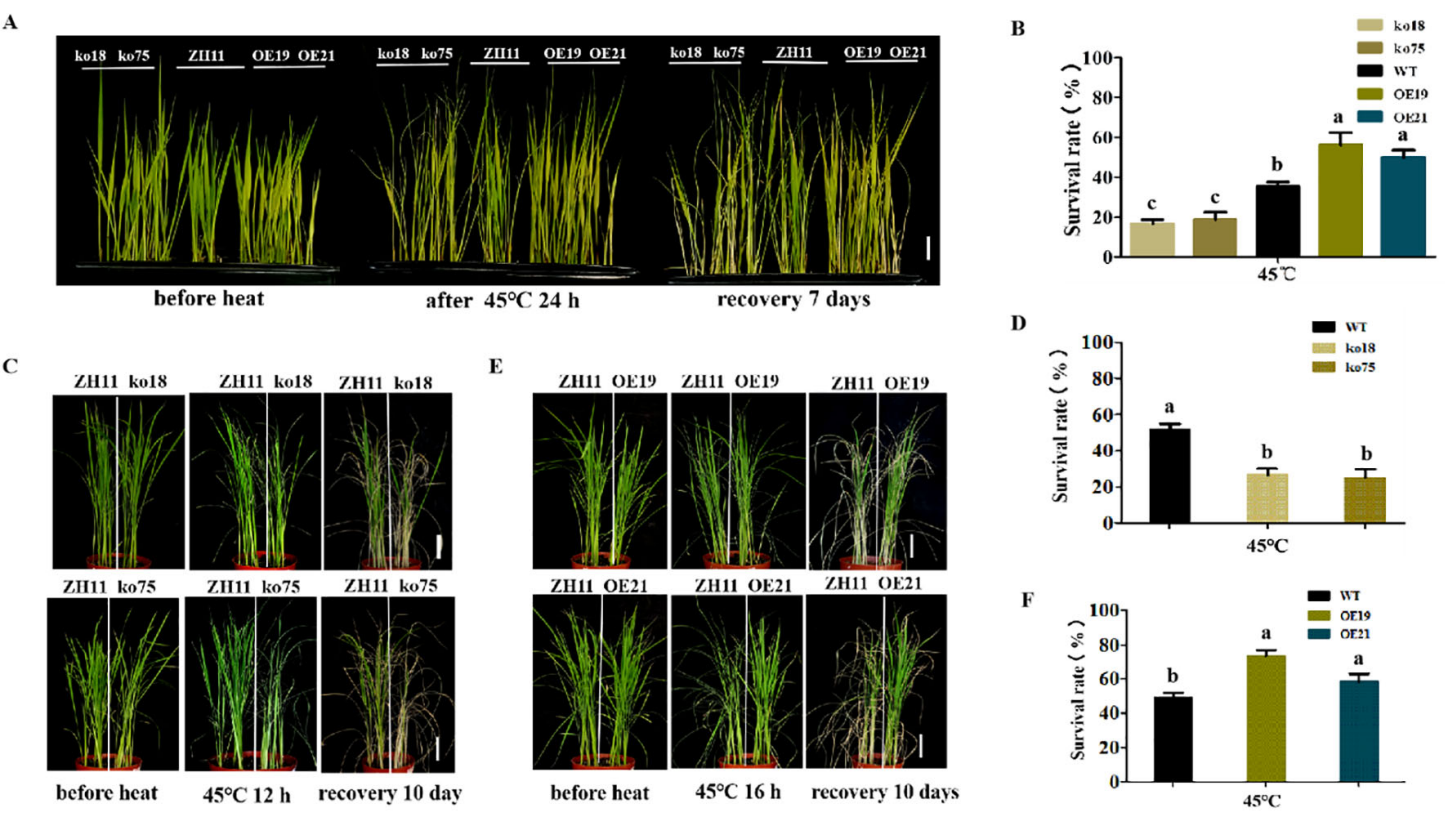
Phenotypic analyses of *OsDUGT1* transgenic rice under heat stress **(A, B)** Phenotypes and survival rates of hydroponically grown rice seedlings after heat treatment. *Osdugt1* knockout lines (ko18, ko75), wild type (ZH11) and *OsDUGT1* overexpressing lines (OE19, OE21) were treated under 45°C for 24h and then recovery for 7 days. The scale bar = 2 cm. **(C–F)** Phenotypes and survival rates of soil-grown rice seedlings after heat treatment. **(C, D)** ko18, ko75 and ZH11 were treated under 45°C for 12h and then recovery for 10 days. **(E, F)** OE19, OE21 and ZH11 were treated under 45°C for 16h and then recovery for 10 days. Significant differences in **(B, D, F)** were analyzed by one-way ANOVA with Tukey’s test (P < 0.05). The scale bar = 5cm. The average number with the same letter (a, b, c, d) in Tukey's test indicates no significant difference at P < 0.05.

Using the two mutant lines (ko18, ko75) and two overexpression lines (OE19, OE21), we investigated the potential function of *OsDUGT1* in response to high temperature for the hydroponically grown rice and soil-grown rice. We discovered that mutants ko18 and ko75 consistently displayed a more susceptible phenotype to the heat stress with reduced survival rates in comparison to wild type ZH11, regardless of whether the rice was cultivated hydroponically or in soil. In contrast to the wild type, the overexpression lines OE19 and OE21 consistently exhibited the improved resistance to heat stress and provided greater survival rates (**Figure 2**). These findings showed that *OsDUGT1* confers heat tolerance on rice and has a beneficial regulatory role in the response to high temperatures.

**Figure 3:**
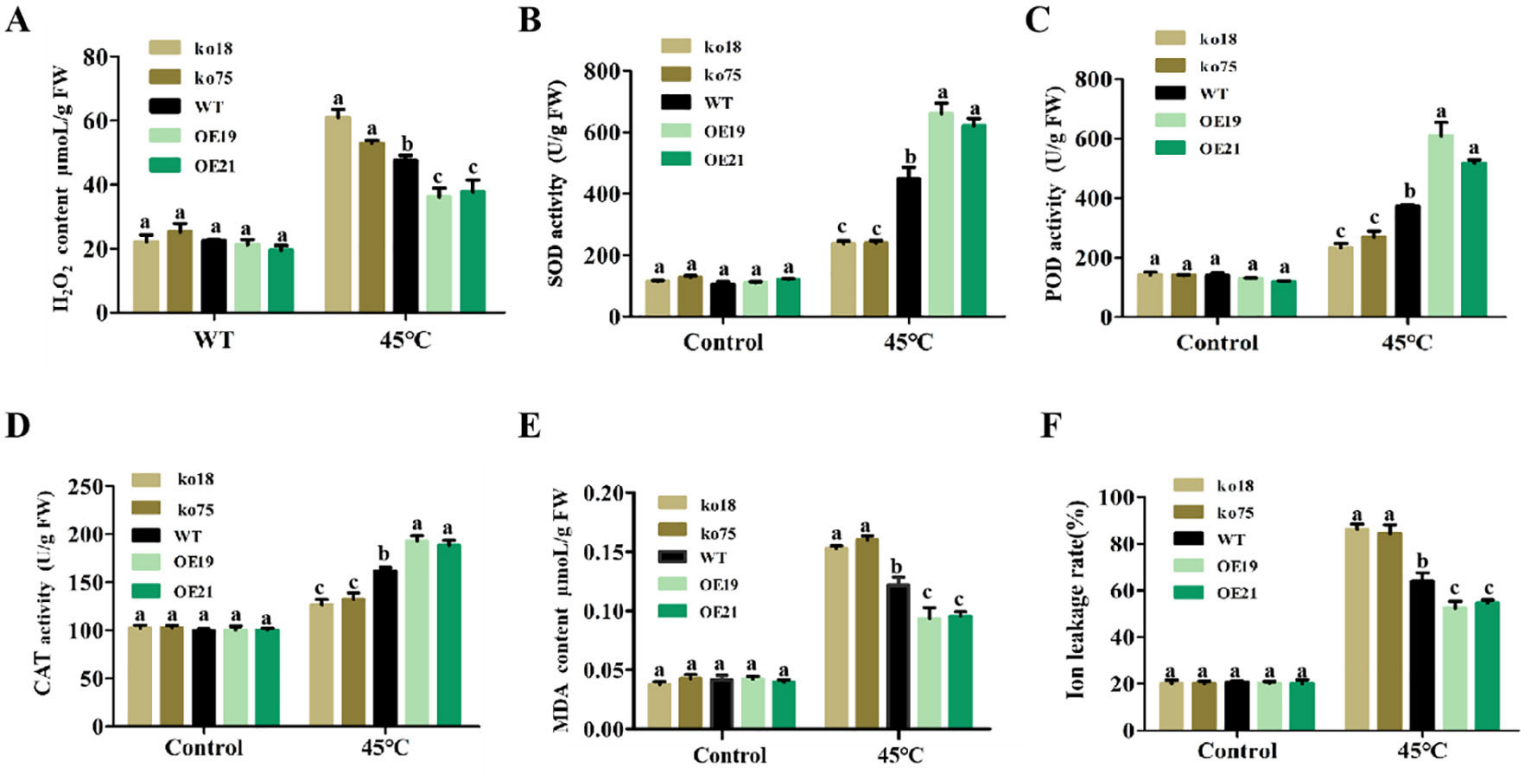
Determination of physiological indexes in *OsDUGT1* transgenic rice plants under control and heat stress condition. **(A–F)** indicate the H_2_O_2_ content, SOD, POD and CAT activity, MDA content, and ion leakage, respectively. Significant differences were analyzed by one-way ANOVA with Tukey’s test (P < 0.05). The average number with the same letter (a, b, c, d) in Tukey's test indicates no significant difference at P < 0.05.

When plants are consistently exposed to severe heat stress, intracellular ROS levels will increase significantly, thus breaking the ROS homeostasis and causing oxidative damage (Zhao et al., 2018b). To find out how *OsDUGT1*’s physiological mechanism affect rice’s ability to withstand heat, we measured H_2_O_2_ content and discovered that while there were no appreciable differences between the mutant lines, wild type lines and overexpression lines under the control condition, however, the H_2_O_2_ content in *Osdugt1* knockout mutant lines after high temperature stress was significantly higher compared to ZH11 and overexpression lines, increased by 0.2 and 0.5 folds, respectively (**Figure 3A**). These differences were statistically significant. This suggests that at high temperatures, the loss of *OsDUGT1* gene function causes a large amount of ROS accumulation. Besides, using diaminobenzidine (DAB) and nitroblue tetrazolium (NBT) staining techniques, we further explored the impact of *OsDUGT1* in improving antioxidant capacity. According to the staining results, the rice leaves of the ko18 and ko75 mutant seedlings stained more deeply than those of ZH11 when exposed to high temperature stress, while the leaves of the OE19 and OE21 transgenic seedlings stained lighter than those of ZH11; however, there was no discernible difference between the transgenic and ZH11 seedlings’ leaves under normal circumstances (**Supplementary Figure S2**). To cope with oxidative stress, plants often rely on enzymatic or non-enzymatic antioxidant defenses. Antioxidant enzymes including catalase (CAT), superoxide dismutase (SOD), and peroxidase (POD) are thought to be the most effective kinds of defense. After heat stress, our measurements showed that the overexpression lines’ CAT, SOD, and POD enzyme activities were significantly higher than ZH11, whereas those enzyme activities in mutant lines were lower than ZH11 (**Figures 3B–D**). These results suggested that *OsDUGT1* responds to heat stress by eliminating reactive oxygen species to a greater degree, thus alleviating plant damage.

Usually, high-temperature increases membrane fluidity, causes changes in cell membrane permeability, and results in a significant loss of cellular electrolytes, which inhibits cellular function and reduces heat resistance. On the other hand, membrane lipid peroxidation in plants is detected by measuring malondialdehyde (MDA). MDA is a widely used marker of oxidative lipid injury caused by environmental stress. Because MDA concentration and ion leakage rate represent the degree of cell membrane damage (Prerostova et al., 2022), we additionally assessed their changes. Following 45°C treatment, it was observed that the *Osdugt1* mutant lines’ MDA content and ion leakage rate were much higher than those of the wild type, whereas the overexpression lines’ values were significantly lower (**Figures 3E, F**). These results suggested that *OsDUGT1* confers heat tolerance on rice at least through the increased ROS scavenging and the cellular membrane protection.

### *OsDUGT1* ectopic expression in *Arabidopsis* improves heat tolerance

**Figure 4:**
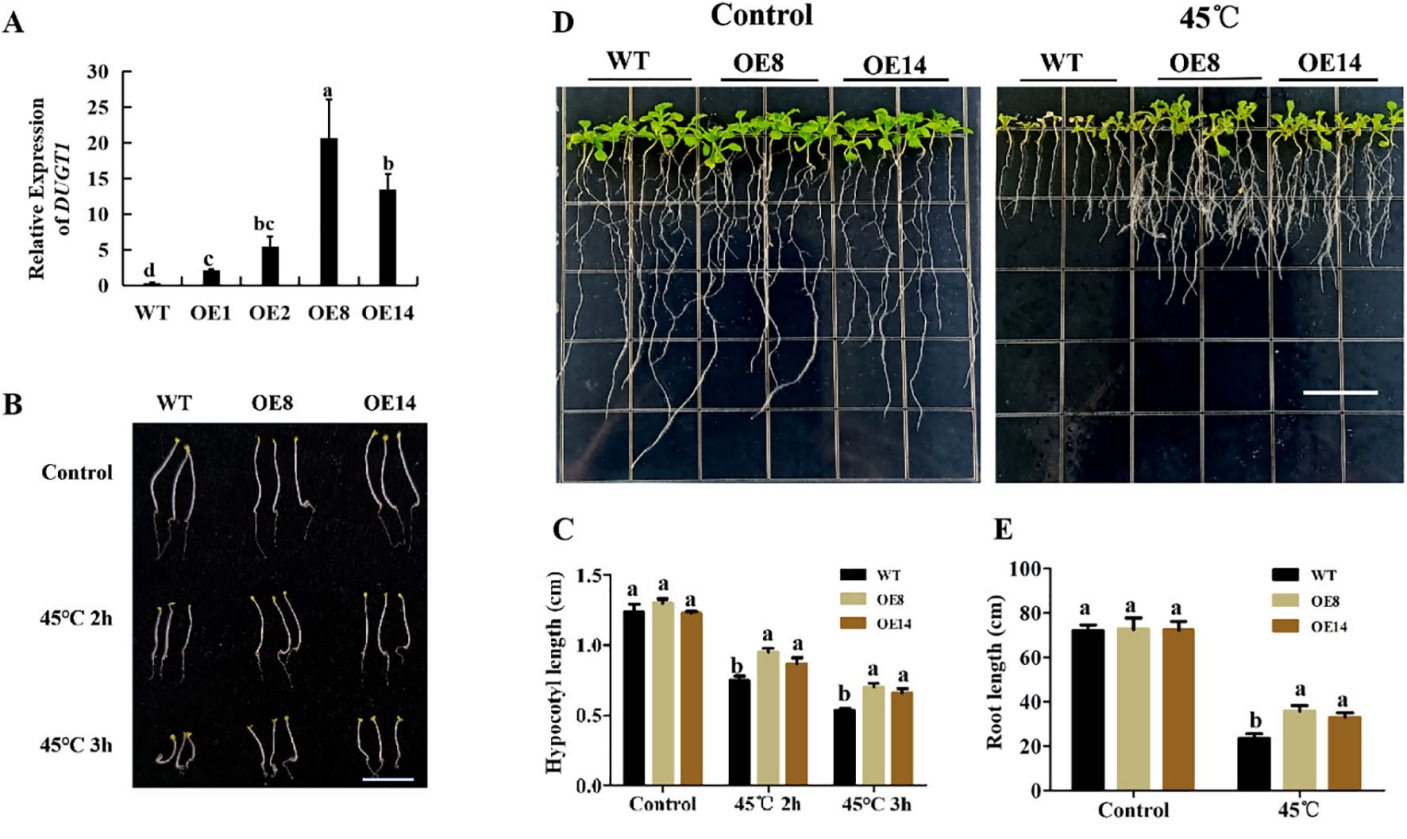
Hypocotyl and root growth of *OsDUGT1-*overexpressing Arabidopsis plants under heat stress condition. **(A)** qRT-PCR analysis showed the expression level of *OsDUGT1* in *Arabidopsis thaliana*. Values are normalized against *AtACTIN2*. **(B, C)** Hypocotyl growth and hypocotyl length after heat stress treatment. The scale bar = 1cm. **(D, E)** Root growth and root length after heat stress treatment. The scale bar =1cm. Significant differences in **(C, E)** were analyzed by one-way ANOVA with Tukey’s test (P < 0.05). The average number with the same letter (a, b, c, d) in Tukey's test indicates no significant difference at P < 0.05.

**Figure 5:**
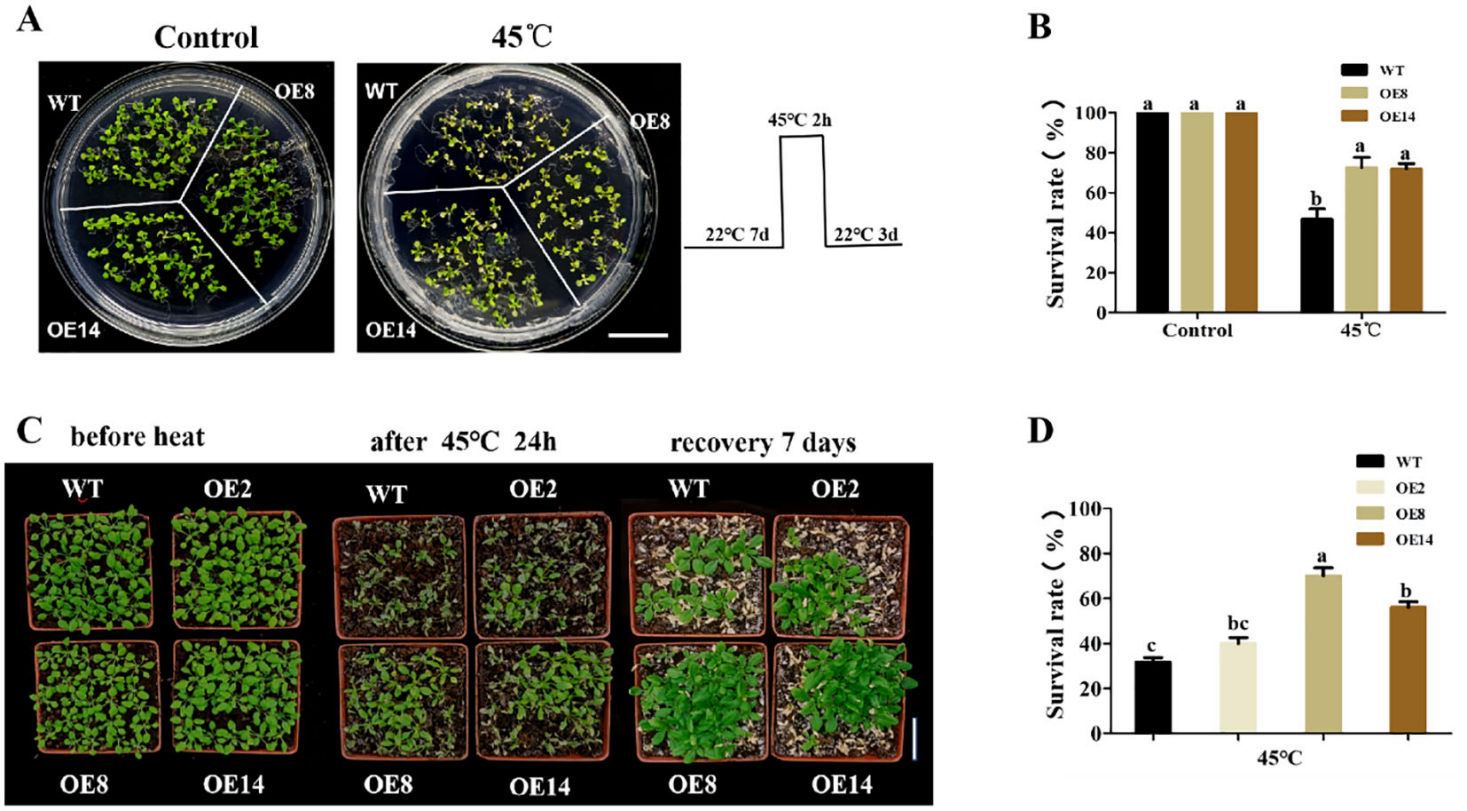
Growth phenotypes and survival rates of *OsDUGT1-*overexpressing Arabidopsis plants under heat stress condition. **(A, B)** Growth phenotypes and survival rates of *OsDUGT1-*overexpressing Arabidopsis seedlings grown on media after heat stress treatment. The scale bar = 2 cm. **(C, D)** Growth phenotypes and survival rates of *OsDUGT1-*overexpressing Arabidopsis plants grown on soil after heat stress treatment. The scale bar = 5 cm. Data are means ± SD. Significant differences in **(B, D)** were analyzed by one-way ANOVA with Tukey’s test (P < 0.05). The average number with the same letter (a, b, c, d) in Tukey's test indicates no significant difference at P < 0.05.

To further verify the function of *OsDUGT1* in high temperature response, we heterogeneously expressed this gene in *Arabidopsis thaliana*, and acquired several overexpression lines (OE1, OE2, OE8, OE14) (**Figure 4A**; **Supplementary Figure S1C**). The hypocotyl length of *OsDUGT1* overexpression lines (OE8, OE14) were first measured after heat stress treatment at 45°C. We discovered that after heat stress treatment, the hypocotyl length of overexpression lines was noticeably longer than that of wild-type plants (**Figures 4B, C**). Then, we assessed the transgenic *Arabidopsis*’s root elongation after heat treatment. Similar with the hypocotyl growth, the roots of *OsDUGT1* overexpression lines were significantly longer than those of the wild type, and with much more lateral roots (**Figures 4D, E**). To further investigate whether *OsDUGT1* overexpressing plants can enhance the heat tolerance of *Arabidopsis thaliana*, *OsDUGT1* overexpressing *Arabidopsis* plants grown on media and grown on soil were subjected to heat stress treatment. The results indicated that the growth phenotype and survival rate of plants overexpressing *OsDUGT1* were obviously superior to those of the wild type (**Figure 5**). These results further demonstrated the function of *OsDUGT1* gene in enhancing plant tolerance to heat stress.

### *OsDUGT1* has broad-spectrum flavonoid glycosyltransferase activity and modulates the accumulation pattern of flavonoids in rice

**Figure 6:**
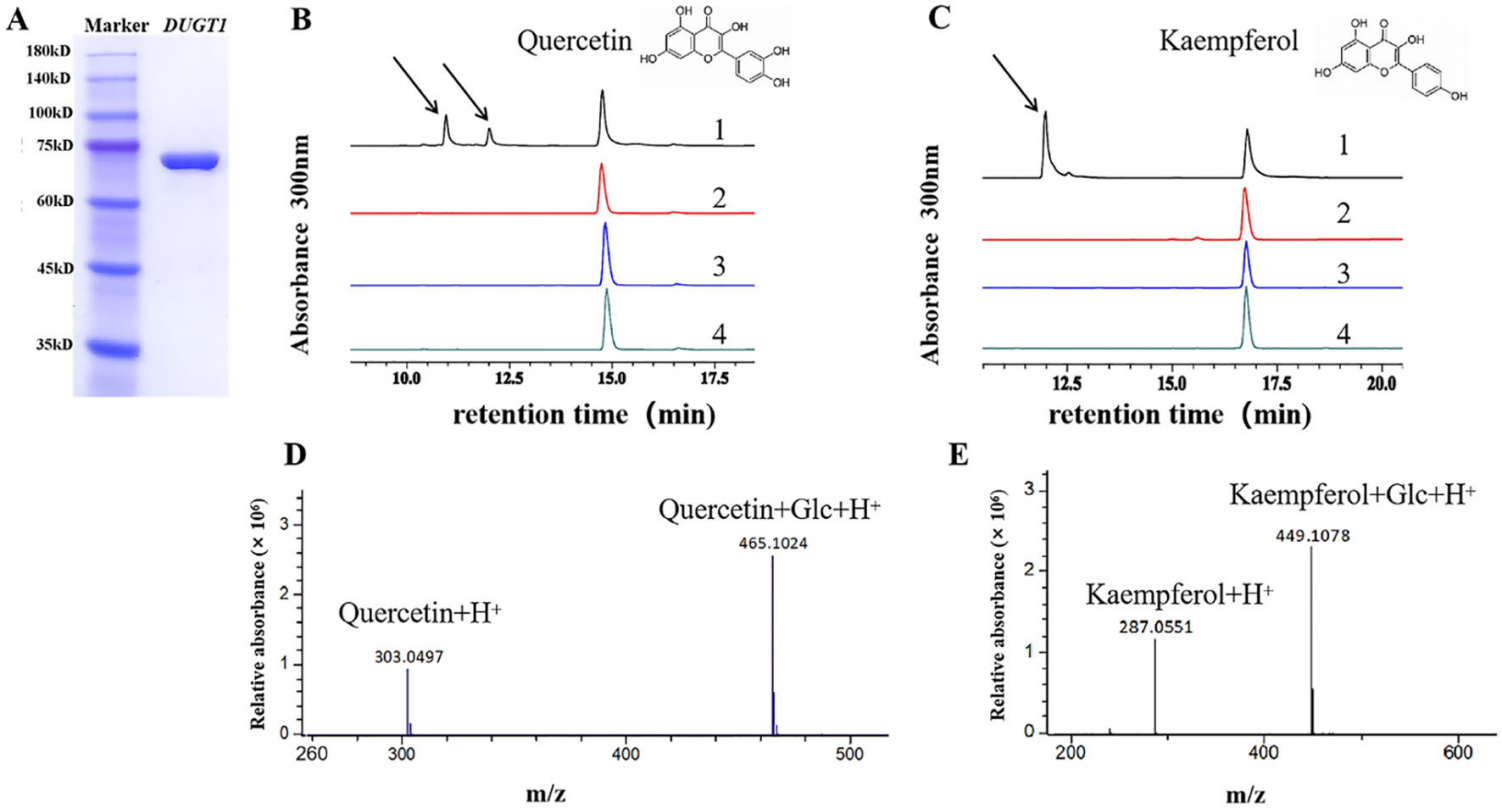
OsDUGT1 catalyzes the glycosylation of quercetin and kaempferol. **(A)** SDS-PAGE detection of the purified recombinant OsDUGT1-GST fusion protein. **(B, C)** The glycosyltransferase activity of OsDUGT1 toward flavonoids was determined by HPLC analysis. 1: putative glucosides of flavonoids were formed in reaction mixture. Arrows indicates the formed glucosides of flavonoids. 2: negative control of reaction with inactivated OsDUGT1. 3: negative control of reaction without sugar donor. 4: negative control of reaction without OsDUGT1 enzyme and sugar donor. **(D, E)** LC–MS/MS analysis confirmed the formation of flavonoid glucosides by OsDUGT1 catalytic activity.

In order to know the molecular mechanism of *OsDUGT1* in enhancing plant tolerance to heat stress, we analyzed the glycosyltransferase activity of OsDUGT1 by screening its substrates *in vitro*. The OsDUGT1 fusion protein with GST tag was first expressed in *E.coli* and purified (**Figure 6A**). Using the purified OsDUGT1-GST fusion protein as recombinant enzyme and UDP-glucose as sugar donor, a variety of candidate substrates were detected, including flavonoids, phenolic acids, plant hormones and other compounds. We found that *OsDUGT1* had a clear glycosyltransferase activity toward ten flavonoids (**Figures 6B, C** and **Supplementary Figure S3**), but no catalytic activity toward other compounds tested here was detected. Thus, these results suggested that the catalytic activity of OsDUGT1 was likely broad spectrum but specifically towards flavonoid group. In order to verify the glycosylation activity of OsDUGT1, the catalytic products of two typical flavonoids, quercetin and kaempferol, were subjected to LC-MS/MS analysis. Experimental results showed that the reaction products of quercetin gave the dominant ion peaks m/z 465.1024 [quercetin+Glc+H^+^] and the reaction products of kaempferol gave the dominant ion peaks m/z 449.1078 [kaempferol+Glc+H^+^] (**Figures 6D, E**). These results are in good agreement with the anticipated kaempferol glucoside and quercetin glucoside, respectively. Therefore, we concluded that OsDUGT1 has glycosylation activity on flavonoids.

**Figure 7:**
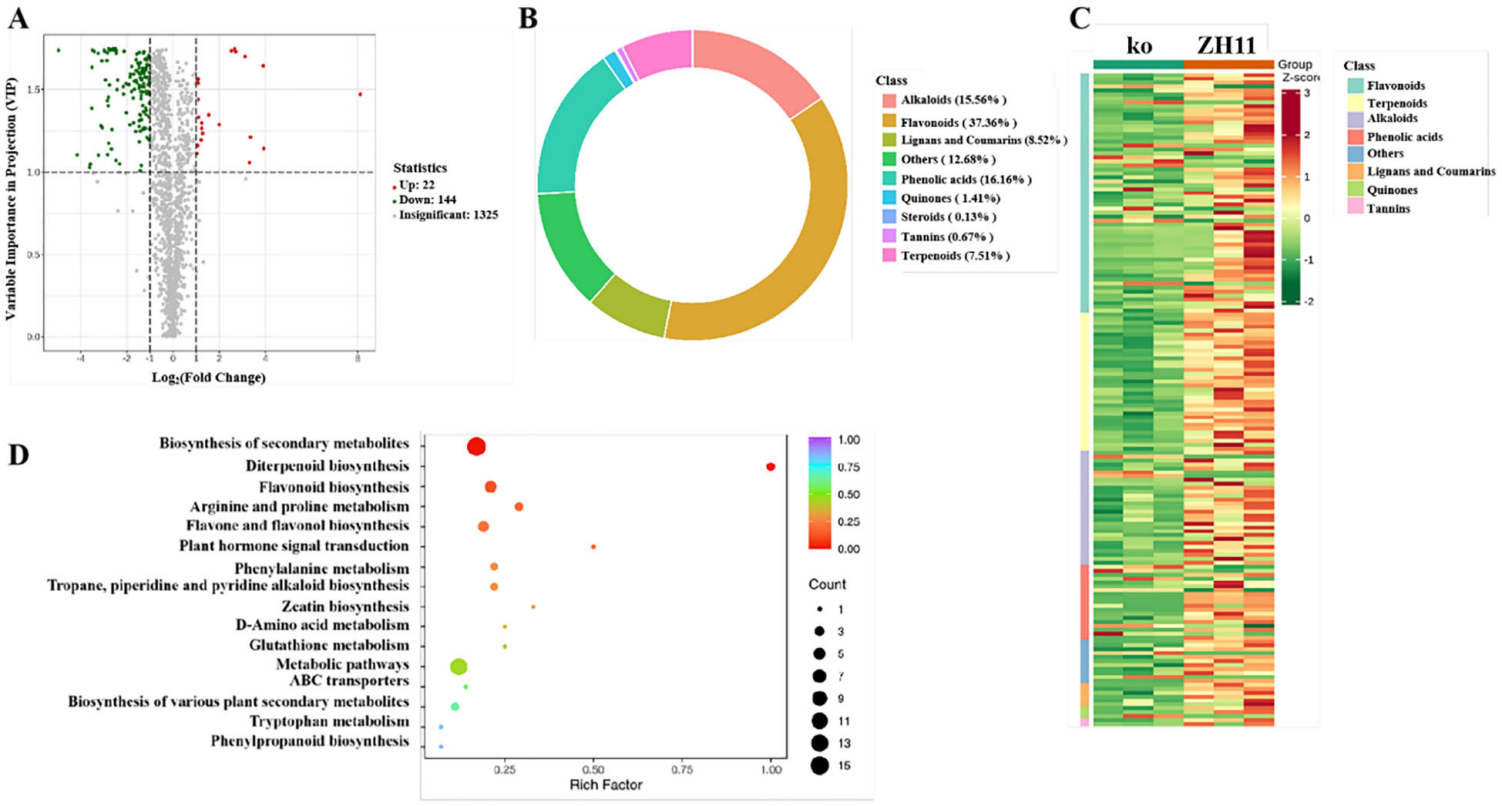
Analysis of differential secondary metabolome of *Osdugt1* knockout mutants (ko) vs. wild type (ZH11) under heat stress. **(A)** Volcano plots comparing the metabolomic of *Osdugt1*-knockout mutants with wild type under 45°C treatment for 24 h The green, gray, and red dots represent reduced, non-different, and increased metabolites, respectively. Fold change ≥ 2, P-value ≤ 0.05. **(B)** Pie chart showing that flavonoids were the largest portion in differential metabolites (37%). **(C)** Heat map clustering analysis of differential metabolites. **(D)** The bubble chart depicting the KEGG enrichment of all differential metabolites in ko vs. ZH11.

To further explore the potential *in vivo* effects and metabolic adjustments resulted by OsDUGT1, we adopted metabolomics analysis to find out the metabolite differences between *Osdugt1* mutants (ko) and wild type ZH11 under high temperature stress. 1491 metabolites were detected and quantified successfully by LC-MS/MS, among which 166 were significantly different, including 144 decreased and 22 increased metabolites in mutants (**Figure 7A**). By cluster analysis, the metabolites with known differences were separated into eight major groups (flavonoids, terpenes, alkaloids, phenolic acids, lignans and coumarins, quinines, tannins, and various others) (**Figures 7B, C**). It is worth pointing out that the most significant changes and the largest portion in differential metabolites were flavonoids, and their contents in knockout mutants were obviously less than that in wild type (**Figures 7B, C**; **Supplementary Table S2**). The KEGG enrichment of all differential metabolites in ko vs ZH11 indicated that the differential metabolites were focused on biosynthesis of secondary metabolites, flavonoid biosynthesis, flavones and flavonol biosynthesis, etc. (**Figure 7D**). It is well-known that flavonoids in planta are mainly in the form of glycosides. Our metabolome analysis indicated that OsDUGT1 can affect the flavonoid metabolism in rice, leading to a notable reduction in the accumulation levels of flavonoid glycosides and total flavonoids in *OsDUGT1* loss-of-function mutants under high temperature stress. Clearly, these metabolic changes and adjustments were highly relevant to the enzyme activity of OsDUGT1 identified here.

### Transcriptomic analysis of the *Osdugt1* knockout mutants

**Figure 8:**
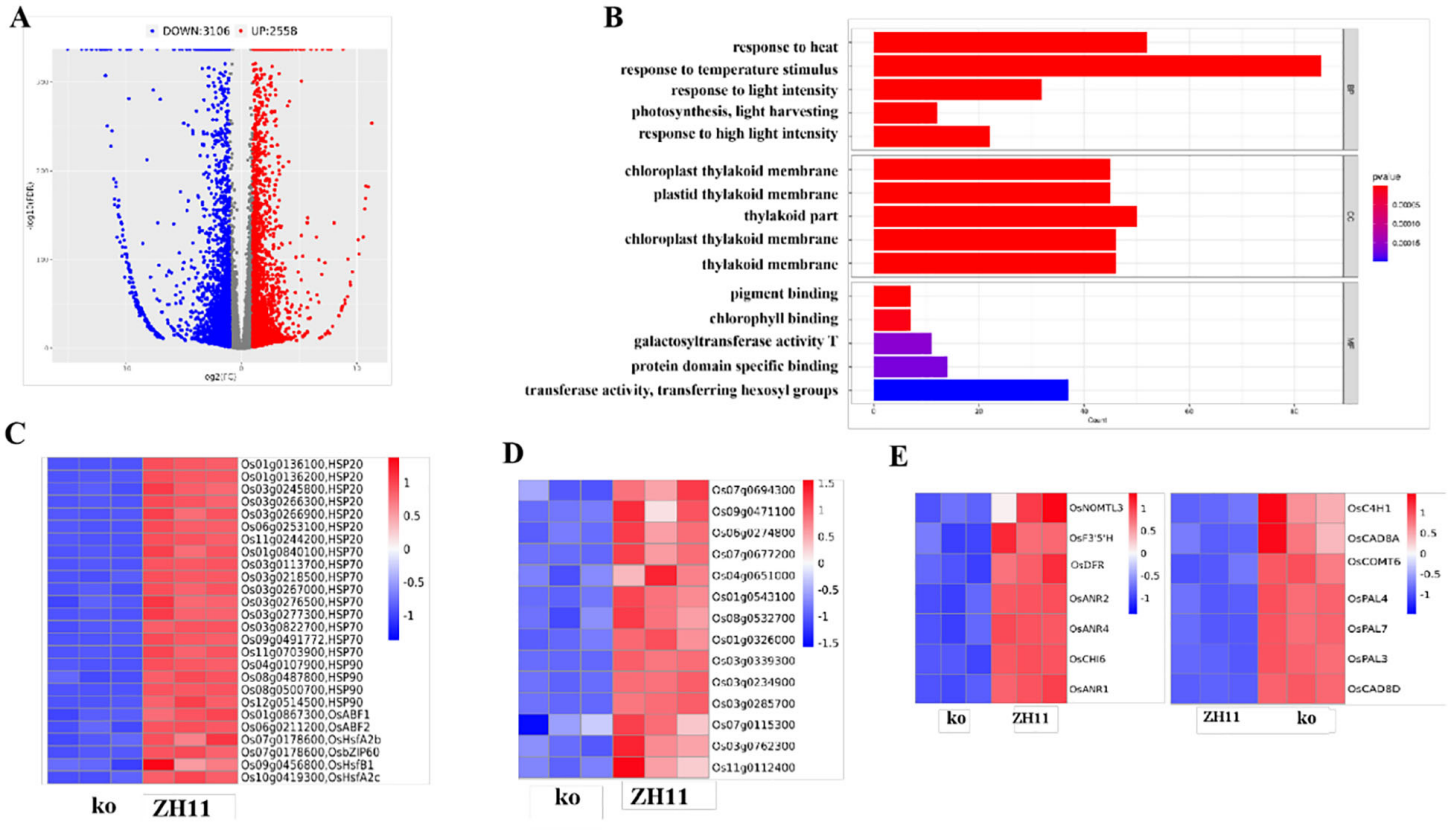
Analysis of differentially expressed genes of *Osdugt1* knockout mutants (ko) vs. wild type (ZH11) under heat stress. **(A)** Volcano plots comparing the transcriptomes of the *Osdugt1*-knockout mutants with wild type under 45°C treatment for 24 (h) The blue, gray, and red dots represent downregulated genes, non-differentially expressed genes, and upregulated genes, respectively. **(B)** The bar chart depicting the GO enrichment of DEGs downregulated in ko vs ZH11. **(C)** Heatmap of heat stress resistance-related genes in GO analysis, showing their transcriptional abundance in the *Osdugt1*-knockout mutants. **(D)** Heatmap of genes associated with ROS scavenging in GO analysis. **(E)** Heatmap of genes associated with flavonoid synthesis and phenylalanine synthesis in KEGG analysis. **(C-E)** The colored bar indicates log2 (fold change) values. The red color represents up-regulation, blue color represents down-regulation.

To know the subsequent effects following the metabolic changes and adjustments by OsDUGT1 in high temperature response of rice, we further performed transcriptomic analysis using RNA-seq technique for the *Osdugt1* mutants and wild type under heat treatment condition. Totally, 5664 differentially expressed genes (DEGs) were found (3106 downregulated and 2558 upregulated) (**Figure 8A**; **Supplementary Figure S4A**).

According to gene ontology (GO) analysis, DEGs were shown to be enriched in molecular function, cellular component, and biological process (**Figure 8B**; **Supplementary Figure S4B**). In detail, those down-regulated DEGs for biological process were enriched in response to heat (GO:0009266), response to temperature stimulus (GO:0009266), response to stress (GO:0006950). As is well known, the genes for the HSF and HSP are essential for the plant’s reaction to heat stress. In *Osdugt1* knockout mutants, a higher proportion of heat stress-responsive genes, such as heat shock proteins (Hsp20, Hsp70 and Hsp90) and transcription factors related to heat stress, were down-regulated (**Figure 8C**), which well matched up with the heat sensitive phenotype of mutant lines. Intriguingly, 14 ROS scavenging-related genes associated with response to oxidative stress (GO:0006979) were also significantly down-regulated in *Osdugt1* knockout mutants (**Figure 8D**), which was well in line with the excessive ROS accumulation observed in mutant lines. We also observed those DEGs associated with chloroplast structure and function.

According to KEGG enrichment analysis of DEGs, we obtained 20 highly enriched terms in *Osdugt1* knockout mutants (**Supplementary Figure S4C**). We found that several genes related to flavonoid pathway and anthocyanin pathway were downregulated in knockout mutants, such as *OsCHI*, *OsF3’5’H*, *OsNOMT*, and *OsDFR* (**Figure 8E**). However, several genes associated with lignin synthesis in the phenylalanine pathway (*OsPAL*, *OsC4H*, *OsCOMT*, *OsCAD*) were upregulated in *Osdugt1* knockout mutants (**Figure 8E**). These results suggested that knockout of *OsDUGT1* might lead to a redirection of metabolic flux from flavonoid metabolism. This may be a reason why the content of flavonoids was reduced in the mutant rice.

## Discussion

Rice is an important food crop, and its yield has been seriously affected by the global temperature rise (Aghamolki et al., 2014; Zhao et al., 2017; Zenda et al., 2022; Ma et al., 2023b). Therefore, it is necessary to discover and explore some resilience genes to cope with the changeable climate change (Khan et al., 2019; Wu et al., 2022; Chang et al., 2024). In this study, we found that the *OsDUGT1* gene plays an important role in enhancing rice tolerance to heat, which was demonstrated by *OsDUGT1* overexpression and knockout in rice, and by transgenic *Arabidopsis*. These results revealed the pivotal role of *OsDUGT1* in adaptation of plants to live on land, suggesting that *OsDUGT1* can be used as alternative strategy for future agricultural heat-resistant crop breeding.

When plants are subjected to severe heat stress, the dynamic balance of ROS will be destroyed, resulting in the accumulation of a large number of ROS, which will cause oxidative damage and disturb growth and development of plants (Lohani et al., 2020; Qi and Wu, 2022). ROS can be eliminated from cells in two main ways: first, by enzyme protection systems like SOD, CAT, and POD; second, by non-enzymatic protection using antioxidants such flavonoids, ascorbic acid, and glutathione (Panda et al., 2024). Previous research showed that as a special secondary metabolite, flavonoids are crucial for plant to resist to biotic and abiotic stresses (Zhang et al., 2021; Ramaroson et al., 2022). An important feature of flavonoids is acting as an effective antioxidant, which can shield plant cells from oxidative damage brought on by abiotic stressors like UV, salt, drought, cold, and heat (Li et al., 2017; Zhan et al., 2019; Shen et al., 2022). For example, it was showed that *OsUGT706D1* and *OsUGT707A2* can catalyze kaempferol, apigenin, and tricin to form flavonoid glycosides. These O-glycosylated flavones can reduce the accumulation of superoxide anion radical in the leaves, and thus the ability of rice to resist UV stress was enhanced (Peng et al., 2017). By promoting flavonoid accumulation and antioxidant activity, *CrUGT87A1* contributes significantly to *Carex rigescens*’ resilience under the treatment of high salt conditions (Zhang K. et al., 2021). In this study, we identified the enzyme activity of OsDUGT1 and found that it has a broad-spectrum activity of catalyzing flavonoid glycosylation. Our metabolome analysis indicated that the content of flavonoids was significantly reduced in *Osdugt1* knockout mutants compared to the wild type ZH11. Transcriptomic analysis also indicated the downregulation of some genes associated with flavonoid pathway and anthocyanin pathway in mutants. It is worth pointing out that these changes occurred in metabolites and gene expression might be directly related with the enzyme activity of OsDUGT1, if assuming that flavonoid glycosylation executes a positive feedback regulation to flavonoid biosynthesis pathway. Therefore, our findings lend support to the notion that OsDUGT1 can glycosylate flavonoids, increase the content of flavonoids, thereby promote the ability of ROS scavenging and improve the rice heat tolerance. Besides the most significantly changed flavonoids, we also found the changes in terpenes, alkaloids and other metabolites in mutant rice. Because the catalytic activity of UGTs is usually considered as miscellaneous, we can’t exclude the possibility that OsDUGT1 may have the potential to glycosylate other metabolites and participate in other biological processes.

**Figure 9:**
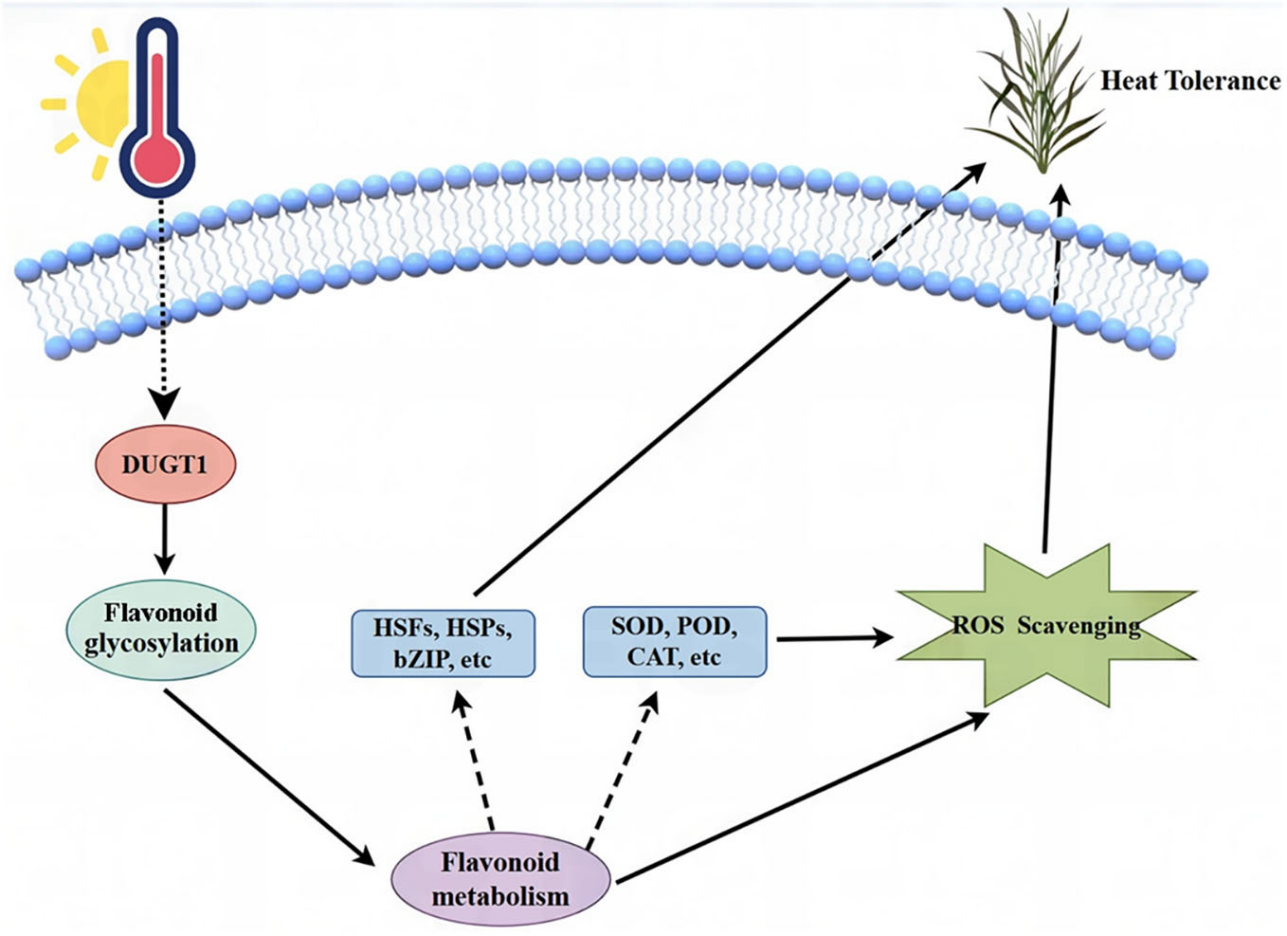
A working model for the function and mechanism of OsDUGT1 in the high temperature stress response.

To gain a deeper understanding of molecular mechanism for OsDUGT1 underlying the heat stress response, we performed RNA-seq on the *Osdugt1* knockout lines and wild type ZH11 after heat stress. Numerous genes with differential expression were identified. We can see that DEGs are mostly involved in heat stress, such as temperature stimulation, membrane transport, and active oxygen scavenging etc. Among these DEGs, there were many *HSPs*, *HSFs*, and ROS scavenging related genes. Moreover, our KEGG analysis also showed that some genes related to flavonoid synthesis pathway and flavonoid metabolism. These results provided new evidence to support that OsDUGT1 can regulate flavonoid metabolism through glycosylation and promote expression of heat stress related genes via some unknown mechanism, thus acting as a key player in the protection of rice against the harmful high temperatures. Integrating the above discussion, we proposed a working model for the function and mechanism of OsDUGT1 in high temperature stress response (**Figure 9**).

In recent years, flavonoids including kaempferol and quercetin have received increasing attention for their health-promoting properties. Animal experiments showed that quercetin and kaempferol have a variety of biological properties in the medical field, such as anticancer, anti-inflammatory, antiviral activities and lower lipid peroxidation (Chen and Chen, 2013; Yang et al., 2023). At the population level, there is increasing evidence that consuming moderate amounts of flavonoids in the daily diet is beneficial to human health (Cassidy and Minihane, 2017). Flavonoids in plants are mainly in the form of glycosides, and glycosides will be more conducive to human absorption (Fan et al., 2022). Given that OsDUGT1 can modulate flavonoid metabolism and increase the accumulation of total flavonoid glycosides, genetic modified food crops with fortified flavonoid glycosides using *OsDUGT1* gene would be well-received for human health in the future.

## Conclusion

To mitigate the effects of high temperature stress on rice growth and yield, functional characterization of genes regulating stress response is essential. In this study, we analyzed the function of rice *OsDUGT1* gene in heat stress adaptation and explored its molecular mechanism. Our experiments revealed that *OsDUGT1* gene plays a vital role in enhancing heat tolerance of plants through glycosylating flavonoids, regulating flavonoid metabolism and promoting expression of heat tolerance related genes. This work not only identifies the physiological significance of *OsDUGT1* gene in high temperature stress adaptation of land plants, but also provides new insight into the understanding of how global metabolic and transcriptional adjustments of land plants are triggered under high temperature challenge. Therefore, this study provides an important theoretic foundation for plant tolerance to heat and a candidate gene for breeding efforts of enhancing crop heat tolerance under the trend of climate warming all over the world.

## Data Availability

The datasets presented in this study can be found in online repositories. The names of the repository/repositories and accession number(s) can be found in the article/**Supplementary Material.**

## Supplementary material

Supplementary Figure 1
*OsDUGT1* expression levels were analyzed by using alternative internal reference gene.

Supplementary Figure 2 DAB and NBT staining and chlorophyll fluorescence measurement.

Supplementary Figure 3 The *in vitro* glycosyltransferase activity of OsDUGT1 on other flavonoids was determined by HPLC analyses.

Supplementary Figure 4 Differentially expressed genes in the *Osdugt1* knockout mutants under heat stress.

Supplementary Table 1 Primers used in the study.

Supplementary Table 2 ko vs ZH11 heatmap class compounds (Flavonoids).

## References

[B1] Aghamolki M. T. Yusop M. K. Zakikhani H. Jaafar H. Z. Aghamolki M. T. K. Kharidah S.. (2014). Heat stress effects on yield parameters of selected rice cultivars at reproductive growth stages. J. Food Agric. Environ. 12, 741–746. doi: 10.3390/ijms222111690

[B2] Bheri M. Pandey G. K. (2018). Protein Phosphatases Meet reactive oxygen species in plant signaling networks. Environ. Exp. Bot. 161, 26–40. doi: 10.1016/j.envexpbot.2018.10.032

[B3] Cassidy A. Minihane A. M. (2017). The role of metabolism (and the microbiome) in defining the clinical efficacy of dietary flavonoids. Am. J. Clin. Nutr. 105 (1). 10.3945/ajcn.116.136051 PMC5183723 27881391

[B4] Chang Y. Fang Y. Liu J. Ye T. Li X. Tu H.. (2024). Stress-induced nuclear translocation of ONAC023 improves drought and heat tolerance through multiple processes in rice. Nat. Commun. 15 (1), 5877. doi: 10.1038/s41467-024-50229-9 38997294 PMC11245485

[B5] Chan-Schaminet K. Y. Baniwal S. K. Bublak D. Nover L. Scharf K. D. (2009). Specific interaction between tomato HsfA1 and HsfA2 treates hetero-oligomeric superactivator complexes for synergistic activation of heat stress gene expression. J. Biol. Chem. 284, 20848–20857. doi: 10.1074/jbc.M109.007336 19491106 PMC2742850

[B6] Chen A. Y. Chen Y. C. (2013). A review of the dietary flavonoid, kaempferol on human health and cancer chemoprevention. Food Chem. 138, 2099–2107. doi: 10.1016/j.foodchem.2012.11.139 23497863 PMC3601579

[B7] Cheng Q. Zhou Y. Liu Z. Zhang L. Song G. Guo Z.. (2015). An alternatively spliced heat shock transcription factor, OsHSFA2dI, functions in the heat stress-induced unfolded protein response in rice. Plant Biol. 17, 419–429. doi: 10.1111/plb.12267 25255693

[B8] Dahuja A. Goswami S. Kumar R. R. Vinutha T. Praveen S. (2022). “Heat Shock Proteins: Catalytic Chaperones Involved in Modulating Thermotolerance in Plants,” in Thermotolerance in Crop Plants. Eds. Kumar R. R. Praveen S. Rai G. K. (Singapore: Springer), 181–194. doi: 10.1007/978-981-19-3800-9_8

[B9] Das A. Pal S. Chakraborty N. Hasanuzzaman M. Adak M. K. (2024). Regulation of reactive oxygen species metabolism and oxidative stress signaling by abscisic acid pretreatment in rice (Oryza sativa L.) seedlings through sub1A QTL under salinity. Plant Stress 11, 100422. doi: 10.1016/j.stress.2024.100422

[B10] Denjalli I. Knieper M. Uthoff J. Vogelsang L. Kumar V. Seidel T.. (2024). The centrality of redox regulation and sensing of reactive oxygen species in abiotic and biotic stress acclimatization. J. Exp. Bot. 75, 4494–4511. doi: 10.1093/jxb/erae041 38329465

[B11] Dolui D. Das A. Hasanuzzaman M. Adak M. K. (2024). Physiological and biomolecular interventions in the bio-decolorization of Methylene blue dye by Salvinia molesta D. Mitch. Int. J. phytoremediation, 1–18. doi: 10.1080/15226514.2024.2412242 39392243

[B12] Dong N. Q. Sun Y. Guo T. Shi C. L. Zhang Y. M. Kan Y.. (2020). UDP-glucosyltransferase regulates grain size and abiotic stress tolerance associated with metabolic flux redirection in rice. Nat. Commun. 11, 1. doi: 10.1038/s41467-020-16403-5 32457405 PMC7250897

[B13] Fan X. Fan Z. Yang Z. Huang T. Tong Y. Yang D.. (2022). Flavonoids—Natural gifts to promote health and longevity. Int. J. Mol. Sci. 23 (4), 2176. doi: 10.3390/ijms23042176 35216290 PMC8879655

[B14] Fang Y. Liao K. Du H. Xu Y. Song H. Li X.. (2015). A stress-responsive NAC transcription factor SNAC3 confers heat and drought tolerance through modulation of reactive oxygen species in rice. J. Exp. Bot. 66, 6803–6817. doi: 10.1093/jxb/erv386 26261267 PMC4623689

[B15] Feng S. Yao Y. T. Wang A. K. (2023). Flavonoids are involved in salt tolerance through ROS scavenging in the halophyte Atriplex canescens. Plant Cell Rep. 43, 1. doi: 10.1007/s00299-023-03087-6 38127154

[B16] Ghorbani A. Tafteh M. Roudbari N. Pishkar L. Zhang W. Wu C. (2021). Piriformospora indica augments arsenic tolerance in rice (Oryza sativa) by immobilizing arsenic in roots and improving iron translocation to shoots. Ecotoxicology Environ. Saf. 209, 111793. doi: 10.1016/j.ecoenv.2020.111793 33360287

[B17] Guihur A. Rebeaud M. E. Goloubinoff P. (2022). How do plants feel the heat and survive? Trends Biochem. Sci. 47, 824–838. doi: 10.1016/j.tibs.2022.05.004 35660289

[B18] Guo X. L. Yuan S. N. Zhang H. N. Zhang Y. Y. Zhang Y. J. Wang G. Y.. (2020). Heat-response patterns of the heat shock transcription factor family in advanced development stages of wheat (*Triticum aestivum* L.) and thermotolerance-regulation by *TaHsfA2–10* . BMC Plant Biol. 20, 364. doi: 10.1186/s12870-020-02555-5 32746866 PMC7397617

[B19] Gupta S. K. Rai K. N. Singh P. Ameta V. L. Gupta S. K. Jayalekha A. K.. (2015). Seed set variability under high temperatures during flowering period in pearl millet. Field Crops Res. 171, 41–53. doi: 10.1016/j.fcr.2014.11.005

[B20] Hassan M. Chattha M. U. Khan I. Chattha M. B. Barbanti L. Aamer M.. (2020). Heat stress in cultivated plants, nature, impact, mechanisms, and mitigation strategies—a review. Plant Biosyst. - Int. J. Dealing All Aspects Plant Biol. 155211 –, 234. doi: 10.1080/11263504.2020.1727987

[B21] Jacob P. Hirt H. Bendahmane A. (2017). The heat-shock protein/chaperone network and multiple stress resistance. Plant Biotechnol. J. 15, 405–414. doi: 10.1111/pbi.12659 27860233 PMC5362687

[B22] Kang Y. Lee K. Hoshikawa K. Kang M. Jang S. (2022). Molecular bases of heat stress responses in vegetable crops with focusing on heat shock factors and heat shock proteins. Front. Plant Sci. 13. doi: 10.3389/fpls.2022.837152 PMC9036485 35481144

[B23] Khan S. Anwar S. Ashraf M. Y. Khaliq B. Sun M. Hussain S.. (2019). Mechanisms and adaptation strategies to improve heat tolerance in rice. A Review. Plants 8, 508. doi: 10.3390/plants8110508 31731732 PMC6918131

[B24] Li Q. Yu H. M. Meng X. F. Lin J. S. Li Y. J. Hou B. K. (2017). Ectopic expression of glycosyltransferase UGT76E11 increases flavonoid accumulation and enhances abiotic stress tolerance in *Arabidopsis* . Plant Biol. 20, 10–19. doi: 10.1111/plb.12627 28902451

[B25] Lohani N. Singh M. B. Bhalla P. L. (2020). High temperature susceptibility of sexual reproduction in crop plants. J. Exp. Bot. 71, 555–568. doi: 10.1093/jxb/erz426 31560053

[B26] Ma Z. Li M. Zhang H. Zhao B. Liu Z. Duan S.. (2023a). Alternative splicing of TaHsfA2-7 is involved in the improvement of thermotolerance in wheat. Int. J. Mol. Sci. 24 (2), 1014. doi: 10.3390/ijms24021014 36674529 PMC9861123

[B27] Ma Z. Lv J. Wu W. Fu D. Lü S. Ke Y.. (2023b). Regulatory network of rice in response to heat stress and its potential application in breeding strategy. Mol. Breed. 43, 68. doi: 10.1007/s11032-023-01415-y 37608925 PMC10440324

[B28] Mackenzie P. I. Owens S. Burchell B. Bock W. Bairoch A. Belanger A.. (1997). The UDP glycosyltransferase gene superfamily, recommended nomenclature update based on evolutionary divergence. Pharmacogenetics 7, 255. doi: 10.1097/00008571-199708000-00001 9295054

[B29] Muhlemann J. K. Younts T. L. B. Muday G. K. (2018). Flavonols control pollen tube growth and integrity by regulating ROS homeostasis during high-temperature stress. Proc. Natl. Acad. Sci. 115, E11188–E11197. doi: 10.1073/pnas.1811492115 30413622 PMC6255205

[B30] Nishizawa A. Yabuta Y. Yoshida E. Maruta T. Yoshimura K. Shigeoka S. (2006). Arabidopsis heat shock transcription factor A2 as a key regulator in response to several types of environmental stress. Plant J. 48, 535–547. doi: 10.1111/j.1365-313X.2006.02889.x 17059409

[B31] Panda S. K. Gupta D. Patel M. Vyver C. V. Koyama H. (2024). Functionality of reactive oxygen species (ROS) in plants: toxicity and control in poaceae crops exposed to abiotic stress. Plants 13 (15), 2071. doi: 10.3390/plants13152071 39124190 PMC11313751

[B32] Peng M. Shahzad R. Gul A. Subthain H. Shen S. Lei L.. (2017). Differentially evolved glucosyltransferases determine natural variation of rice flavone accumulation and UV-tolerance. Nat. Commun. 8 (1), 1975. doi: 10.1038/s41467-017-02168-x 29213047 PMC5719032

[B33] Postiglione A. E. Delange A. M. Ali M. F. Wang E. Y. Houben M. Hahn S. L. (2024). Flavonols improve tomato pollen thermotolerance during germination and tube elongation by maintaining reactive oxygen species homeostasis. Plant Cell 36, 4511–4534. doi: 10.1093/plcell/koae222 39102899 PMC11449072

[B34] Prerostova S. Jarosova J. Dobrev P. I. Hluskova L. Motyka V. Filepova R.. (2022). Heat stress targeting individual organs reveals the central role of roots and crowns in rice stress responses. Front. Plant Sci. 12. doi: 10.3389/fpls.2021.799249 PMC8801461 35111178

[B35] Qi B. Wu C. (2022). Potential roles of stigma exsertion on spikelet fertility in rice. Front. Plant Sci. 13. doi: 10.3389/fpls.2022.983070 PMC9532568 36212346

[B36] Ramaroson M. L. Koutouan C. Helesbeux J. J. Le C. V. Hamama L. Geoffriau E.. (2022). Role of phenylpropanoids and flavonoids in plant resistance to pests and diseases. Molecules 27 (23), 8371. doi: 10.3390/molecules27238371 36500459 PMC9735708

[B37] Sharma L. Priya M. Kaushal N. Bhandhari K. Chaudhary S. Dhankher O. P.. (2019). Plant growth-regulating molecules as thermoprotectants, functional relevance and prospects for improving heat tolerance in food crops. J. Exp. Bot. 71, 569–594. doi: 10.1093/jxb/erz333 31328236

[B38] Shen N. Wang T. Gan Q. Liu S. Wang L. Jin B. (2022). Plant flavonoids, classification, distribution, biosynthesis, and antioxidant activity. Food Chem. 383, 132531. doi: 10.1016/j.foodchem.2022.132531 35413752

[B39] Swindell W. R. Huebner M. Weber A. P. (2007). Transcriptional profiling of arabidopsis heat shock proteins and transcription factors reveals extensive overlap between heat and non-heat stress response pathways. BMC Genomics 8, 125. doi: 10.1186/1471-2164-8-125 17519032 PMC1887538

[B40] Treutter D. (2006). Significance of flavonoids in plant resistance and enhancement of their biosynthesis. Plant Biol. 7, 581–591. doi: 10.1007/s10311-006-0068-8 16388461

[B41] Wu C. Cui K. Fahad S. (2022). Heat stress decreases rice grain weight, evidence and physiological mechanisms of heat effects prior to flowering. Int. J. Mol. Sci. 23, 10922. doi: 10.3390/ijms231810922 36142833 PMC9504709

[B42] Yang L. Gao Y. Bajpai V. K. El-Kammar H. A. Simal-Gandara J. Cao H.. (2023). Advance toward isolation, extraction, metabolism and health benefits of kaempferol, a major dietary flavonoid with future perspectives. Crit. Rev. Food Sci. Nutr. 63, 2773–2789. doi: 10.1080/10408398.2021.1980762 34554029

[B43] Yang Z. R. Mao X. Li R. Z. (2005). Research progress in genetic engineering of plant secondary metabolism. J. Plant Physiol. Mol. Biol. 31, 11–18. 15692173

[B44] Yang L. Wen K. S. Ruan X. Zhao Y. X. Wei F. Wang Q. (2018). Response of plant secondary metabolites to environmental factors. Molecules 23, 762. doi: 10.3390/molecules23040762 29584636 PMC6017249

[B45] Yokotani N. Ichikawa T. Kondou Y. Matsui M. Hirochika H. Oda I. K. (2008). Expression of rice heat stress transcription factor OsHsfA2e enhances tolerance to environmental stresses in transgenic Arabidopsis. Planta 227, 957–967. doi: 10.1007/s00425-007-0670-4 18064488

[B46] Zenda T. Wang N. Dong A. Zhou Y. Duan H. (2022). Reproductive-stage heat stress in cereals, impact, plant responses and strategies for tolerance improvement. Int. J. Mol. Sci. 23 (13), 6929. doi: 10.3390/ijms23136929 35805930 PMC9266455

[B47] Zhan X. Shen Q. Chen J. Yang P. Wang X. Hong Y. (2019). Rice sulfoquinovosyltransferase SQD2.1 mediates flavonoid glycosylation and enhances tolerance to osmotic stress. Plant Cell Environ. 42, 2215–2230. doi: 10.1111/pce.13554 30942482

[B48] Zhang K. Sun Y. Li M. Long R. (2021). CrUGT87A1, a UDP-sugar glycosyltransferases (UGTs) gene from Carex rigescens, increases salt tolerance by accumulating flavonoids for antioxidation in Arabidopsis thaliana. Plant Physiol. Biochem. 159, 28–36. doi: 10.1016/j.plaphy.2020.12.006 33321375

[B49] Zhang Y. M. Yu H. X. Ye W. W. Shan J. X. Dong N. Q. Guo T.. (2021). A rice QTL GS3.1 regulates grain size through metabolic-flux distribution between flavonoid and lignin metabolons without affecting stress tolerance. Commun. Biol. 4 (1), 1171. doi: 10.1038/s42003-021-02686-x 34620988 PMC8497587

[B50] Zhao C. Liu B. Piao S. Wang X. Lobell D. B. Huang Y.. (2017). Temperature increase reduces global yields of major crops in four independent estimates. Proc. Natl. Acad. Sci. 114, 9326–9331. doi: 10.1073/pnas.1701762114 28811375 PMC5584412

[B51] Zhao Q. Zhou L. Liu J. Cao Z. Du X. Huang F.. (2018b). Involvement of CAT in the detoxification of HT-induced ROS burst in rice anther and its relation to pollen fertility. Plant Cell Rep. 37, 741–757. doi: 10.1007/s00299-018-2264-y 29464319

[B52] Zhao Q. Zhou L. Liu J. Du X. Asad M. A. Huang F.. (2018a). Relationship of ROS accumulation and superoxide dismutase isozymes in developing anther with floret fertility of rice under heat stress. Plant Physiol. Biochem. 122, 90–101. doi: 10.1016/j.plaphy.2017.11.009 29202329

