# Supplementary material for: Rice glycosyltransferase OsDUGT1 is involved in heat stress tolerance by glycosylating flavonoids and regulating flavonoid metabolism
Source: Front Plant Sci. 2025 Jan 13;15:1516990. doi: 10.3389/fpls.2024.1516990 (PMC11769934; doi:10.3389/fpls.2024.1516990)
Supplement: Supplementary Figure 1 — OsDUGT1 expression levels were analyzed by using alternative internal reference gene. [file DataSheet1.doc]

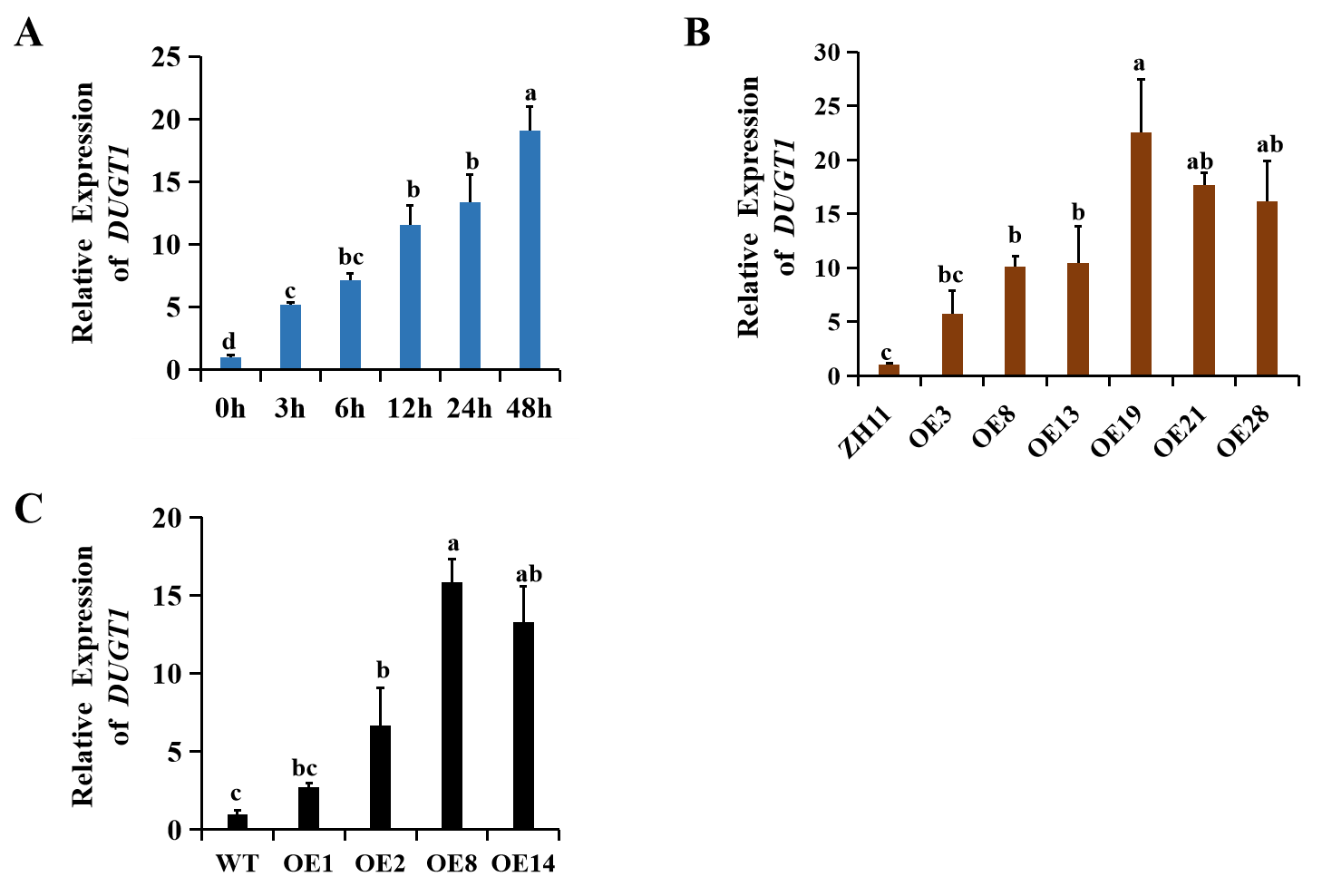


**Figure S1.** ***OsDUGT1* expression levels were analyzed by using alternative internal reference gene**

(**A**) Os*DUGT1* expression patterns of rice under 45℃ treatment through the qRT-PCR analysis. Values are normalized against *OsUBIQ1*. Data are means ± SD.

(**B**) qRT-PCR analysis showing the expression levels of OsDUGT1-overexpressing lines of rice. Values are normalized against *OsUBIQ1*. ZH11 was the wild type rice used in this study. Data are means ± SD.

(**C**) qRT-PCR analysis showed the expression level of *OsDUGT1* in *Arabidopsis thaliana*. Values are normalized against *AtTUB2*. Data are means ± SD.


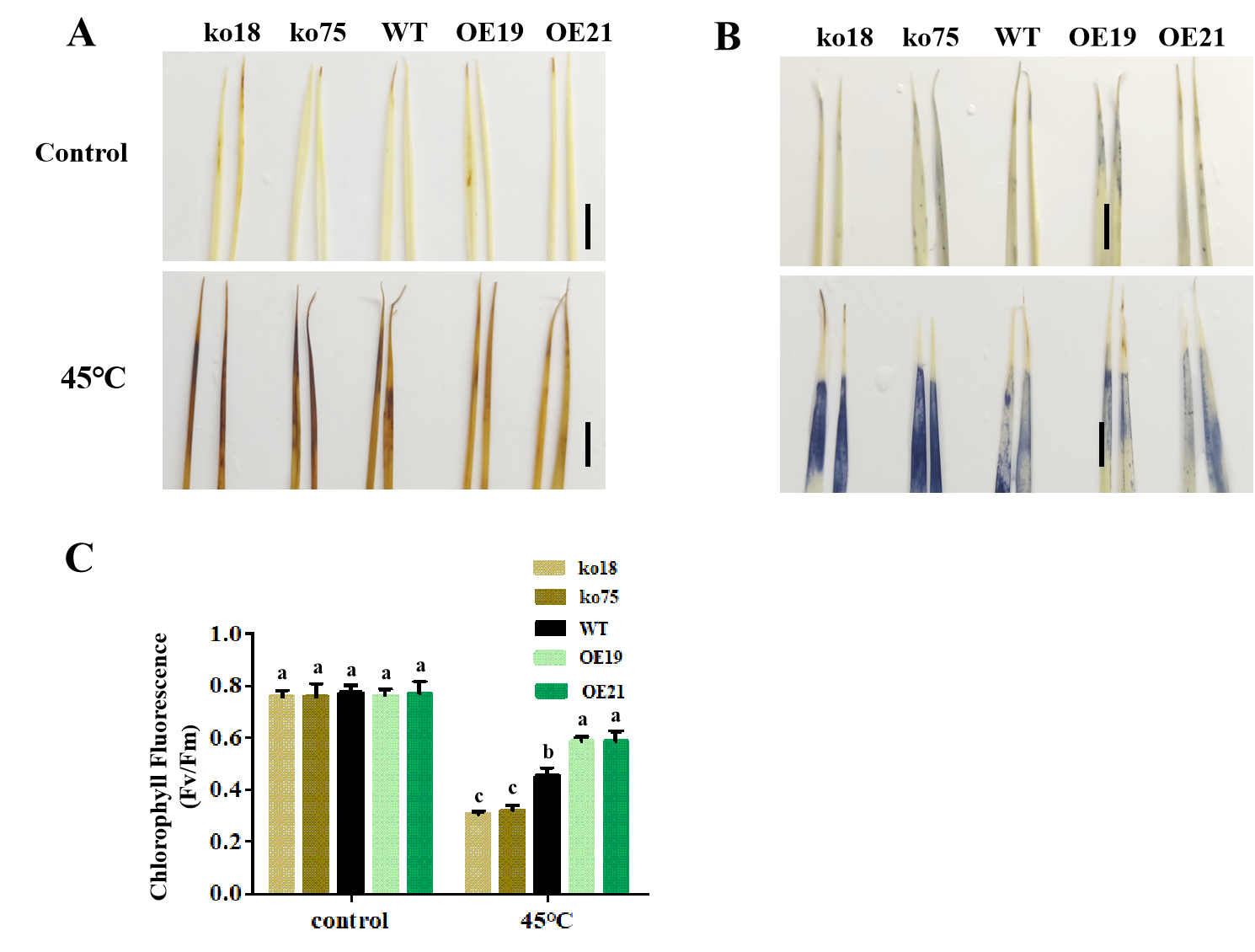


**Figure S2. DAB and NBT staining and chlorophyll fluorescence measurement**

**（A）and (B)** DAB staining and NBT staining. The leaves of *Osdugt1* knockout lines, ZH11, and overexpression lines were treated under control condition (28℃) and heat condition (45℃) for 24h and then subjected to DAB staining (A) and NBT staining (B). Bars = 1 cm.

**(C)** Chlorophyll fluorescence measurement. The leaves of *Osdugt1* knockout lines, ZH11, and overexpression lines were treated under control condition (28℃) and heat condition (45℃) for 24h and then subjected to chlorophyll fluorescence measurement.


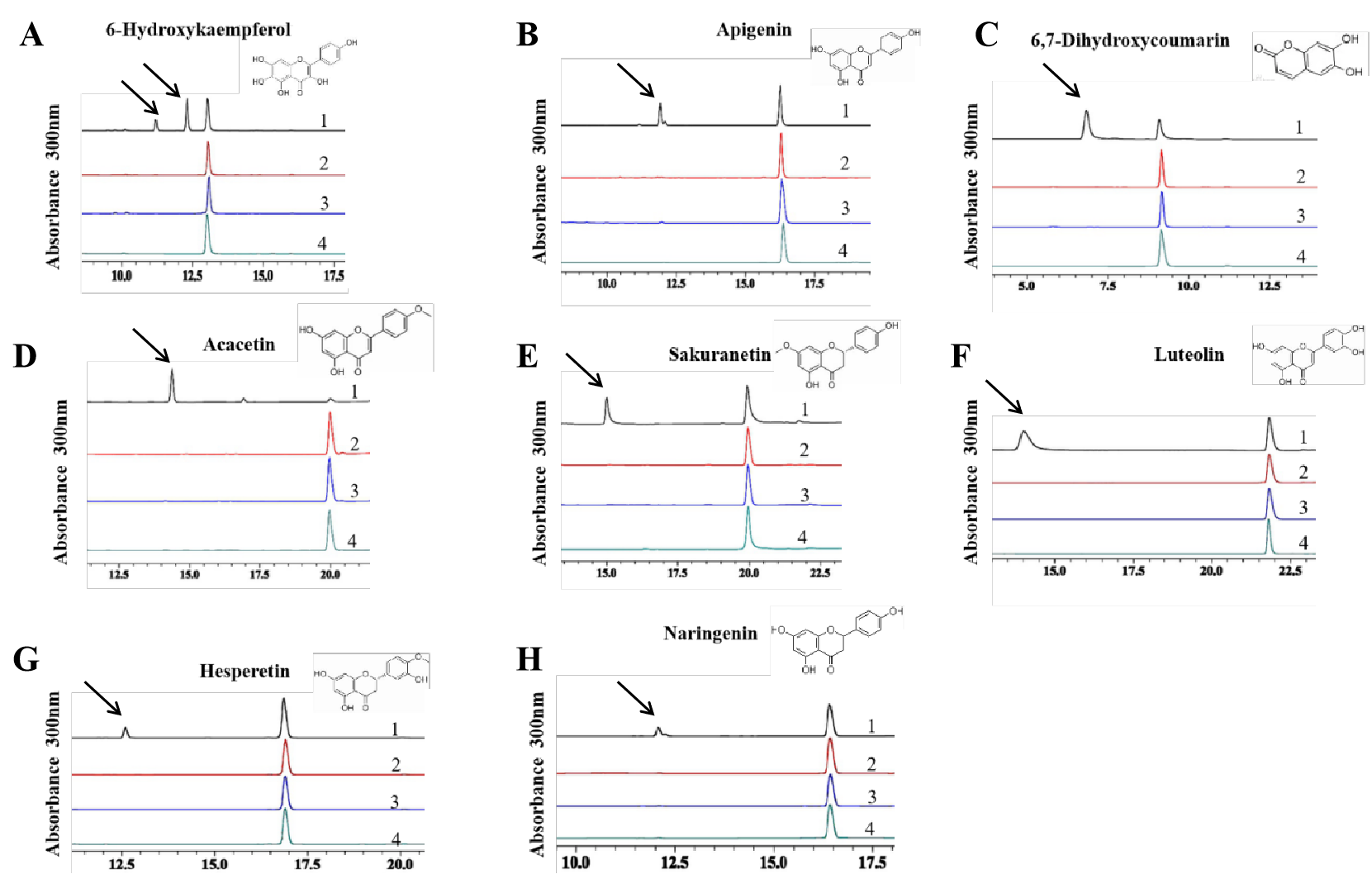


**Figure S3. The in vitro glycosyltransferase activity of OsDUGT1 on other flavonoids was determined by HPLC analyses**

1: putative glucosides of ﬂavonoids were formed in reaction mixture. Arrows indicated the formed glucosides of ﬂavonoids. 2: negative control of reaction with inactivated OsDUGT1. 3: negative control of reaction without sugar donor. 4: negative control of reaction without OsDUGT1 enzyme and sugar donor.


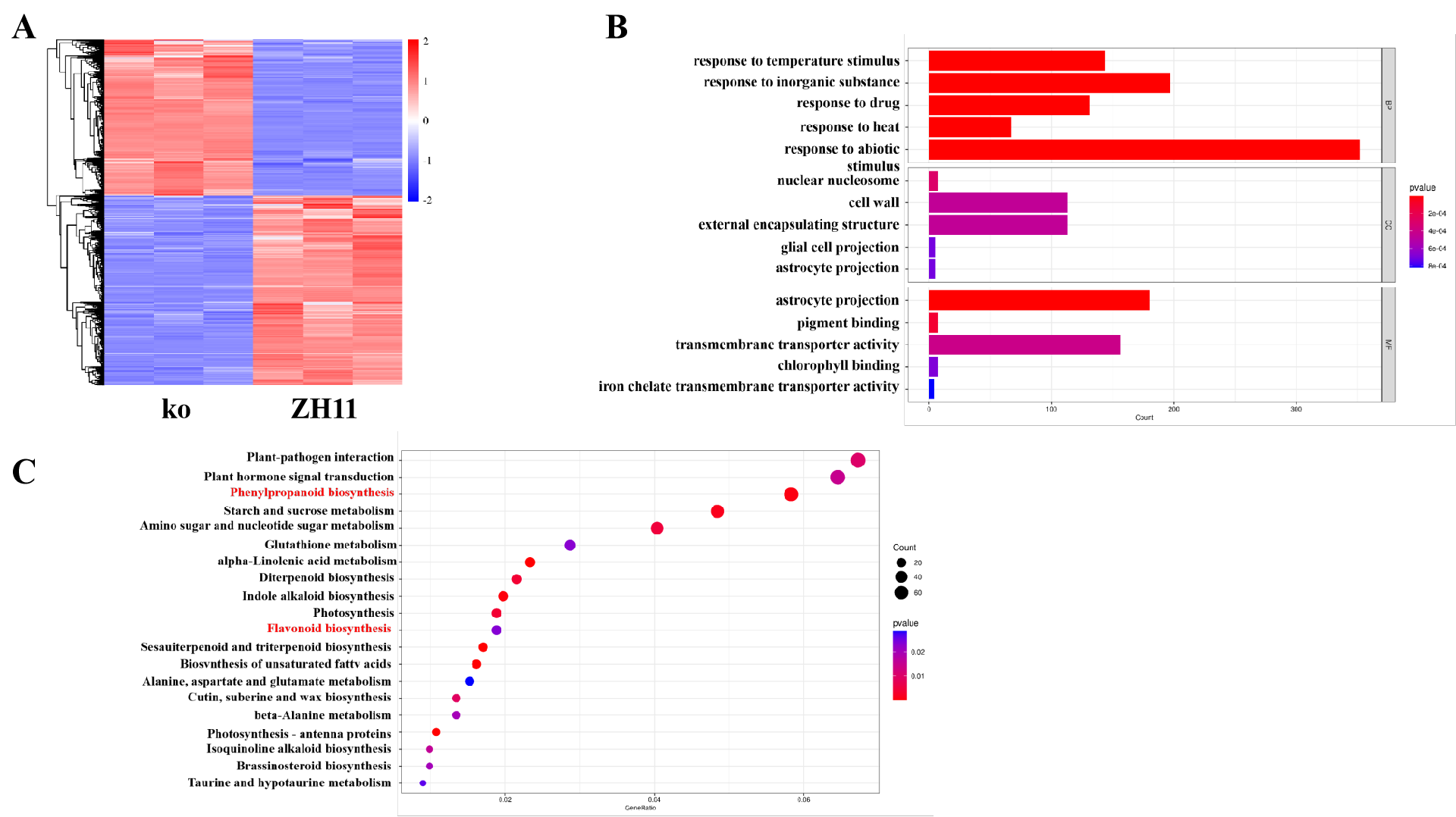


**Figure S4. Differentially expressed genes in the *Osdugt1* knockout mutants under heat stress**

**(A)** Heatmap clustering analysis of all differentially expressed genes. The horizontal axis shows the name of the sample group, each group has three replicates, the vertical axis shows different genes. The colored bar indicates log2 (fold change)≤ 0.5 values , red represents up-regulation, blue represents down-regulation.

1. The bar chart depicting the GO enrichment of all DEGs in ko vs ZH11.
2. The bubble chart depicting the KEGG enrichment of all DEGs in ko vs ZH11.

**Table S1. Primers used in the study**

| **Primer name** | **Primer sequence (5′-3′)** | **Usage** |
| --- | --- | --- |
| qDUGT1-F | ACGGCTTCTTCACTTGGTC | For real-time PCR analysis |
| qDUGT1-R | GCGTCGTTCATCATCTTG |
| qOsACTIN1-F | GGACCCAAGAATGCTAAGCC |
| qOsACTIN1-R | TGGTACCCTCATCAGGCATC |
| qOsUBIQ1-F | GGAGCTGCTGCTGTTCTTGG |
| qOsUBIQ1-R | CACAATGAAAACGGGACACGA |
| qAtACTIN2-F | CATCAGCCGTTTTGAATCTC |
| qAtACTIN2-R | GTAGATCCAGAAAGTTCCTA |
| qAtTUB2-F | GAGCCTTACAACGCTACTCTGTCTGTC |
| qAtTUB2-R | ACACCAGACATAGTAGCAGAAATCAAG |
| DUGT1-taget1 | ACGGCGCTTGCTTCCGAGG | For mutant construction |
| DUGT1-taget2 | GTGGCGCACCATGCTCTCC |
| *dugt1*-F | TCAGGTGCACCATCCTCACCAC | For mutant identification |
| *dugt1*-R | CTGGTCGGCGTCGTTCATCATC |
| DUGT1-pUN1301-F | tgaacgatcgagctcggtaccATGGCTACCATGGATGAGCAGC | For plasmid construction (lowercase letters denoted homologous arm sequences used for plasmid construction) |
| DUGT1-pUN1301-R | gccaaatgtttgaacgatcgagctcTCATACGTCAACAGAGGTCCGG |
| DUGT1-pB121-F | acgggggactctagaggatccATGGCTACCATGGATGAGCAGC |
| DUGT1-pB121-R | cgatcggggaaattcgagctcTCATACGTCAACAGAGGTCCGG |
| DUGT1-pGEX4T-F | gttccgcgtggatccccgATGGCTACCATGGATGAGCAGC |
| DUGT1-pGEX4T-R | ctcgagtcgacccgggaattcTCATACGTCAACAGAGGTCCGG |

**Table S2. Differential metabolites of ko vs. ZH11 (Flavonoids)**

| **Index** | **Compounds** | **Class I** | **ko-1** | **ko-2** | **ko-3** | **ZH11-1** | **ZH11-2** | **ZH11-3** |
| --- | --- | --- | --- | --- | --- | --- | --- | --- |
| MWSHY0113 | Quercetin-3-O-galactoside (Hyperin)* | Flavonoids | 3.22E+05 | 4.14E+05 | 6.62E+05 | 7.72E+05 | 4.39E+06 | 1.08E+07 |
| pmp001309 | 6-Hydroxykaempferol-7-O-glucoside | Flavonoids | 3.17E+05 | 9.10E+05 | 5.38E+05 | 4.32E+05 | 3.54E+06 | 1.26E+07 |
| Lmjp002461 | Quercetin-3-O-neohesperidoside* | Flavonoids | 6.89E+05 | 1.31E+06 | 2.25E+06 | 1.73E+06 | 9.40E+06 | 2.58E+07 |
| Zbhp004510 | Hesperetin-3'-O-glucoside | Flavonoids | 4.10E+05 | 6.68E+05 | 3.95E+05 | 5.33E+05 | 4.08E+06 | 7.68E+06 |
| Lmmp002334 | Quercetin-3-O-rutinoside-7-O-glucoside | Flavonoids | 5.82E+03 | 5.82E+03 | 5.82E+03 | 3.36E+04 | 4.59E+04 | 5.71E+04 |
| Smgp004575 | Quercetin-5-O-β-D-glucoside* | Flavonoids | 3.25E+05 | 3.53E+05 | 8.86E+05 | 9.21E+05 | 4.71E+06 | 5.61E+06 |
| MWSHY0067 | Quercetin-3-O-rutinoside (Rutin)* | Flavonoids | 8.39E+05 | 2.24E+06 | 2.39E+06 | 1.54E+06 | 7.42E+06 | 3.01E+07 |
| Lmdp003286 | Quercetin-3-O-alloside; Isohyperoside* | Flavonoids | 3.81E+05 | 3.95E+05 | 9.84E+05 | 9.58E+05 | 3.99E+06 | 7.29E+06 |
| MWSHY0046 | Quercetin-3-O-glucoside (Isoquercitrin)* | Flavonoids | 3.55E+05 | 4.13E+05 | 9.78E+05 | 1.09E+06 | 4.97E+06 | 6.03E+06 |
| Lmsp004166 | Quercetin-3-O-glucoside-7-O-rhamnoside* | Flavonoids | 7.91E+05 | 1.67E+06 | 2.47E+06 | 1.43E+06 | 1.07E+07 | 2.17E+07 |
| Lmmp002755 | Quercetin-7-O-rutinoside-4'-O-glucoside | Flavonoids | 3.33E+03 | 1.08E+04 | 9.98E+03 | 2.22E+04 | 6.58E+04 | 7.43E+04 |
| Lmmp003091 | Quercetin-3-O-(4''-O-glucosyl)rhamnoside* | Flavonoids | 9.21E+05 | 2.00E+06 | 2.89E+06 | 1.67E+06 | 9.06E+06 | 2.59E+07 |
| Zbsp004301 | Quercetin-7-O-rutinoside* | Flavonoids | 1.26E+06 | 2.07E+06 | 2.67E+06 | 1.36E+06 | 1.17E+07 | 2.02E+07 |
| Zbsp004060 | Apigenin-6-C-xyloside-8-C-arabinoside | Flavonoids | 9.39E+05 | 8.29E+05 | 1.34E+06 | 5.47E+06 | 1.40E+06 | 1.72E+06 |
| Lagp003663 | 7,3',4'-Trihydroxyquercetin glucoside | Flavonoids | 7.70E+04 | 1.08E+05 | 6.34E+04 | 9.54E+04 | 1.81E+05 | 3.84E+05 |
| pme3504 | Formononetin-7-O-glucoside (Ononin) | Flavonoids | 3.00E+04 | 4.27E+03 | 4.27E+03 | 2.82E+04 | 3.00E+04 | 3.62E+04 |
| Zmhp005139 | Tamarixetin-3-O-(6''-malonyl)glucoside | Flavonoids | 3.78E+04 | 3.43E+04 | 3.85E+04 | 9.42E+04 | 7.90E+04 | 8.93E+04 |
| Zbsp004183 | Tricin-5-O-(3'-malonyl)glucoside-7-O-glucoside | Flavonoids | 6.36E+04 | 2.59E+04 | 7.03E+04 | 1.50E+05 | 1.45E+05 | 7.03E+04 |
| Lahp003481 | Eriodictyol apiosyl glucoside | Flavonoids | 1.30E+04 | 3.26E+04 | 1.90E+04 | 3.12E+04 | 5.43E+04 | 5.86E+04 |
| Zmxp005470 | Tricin-7-O-(6''-O-malonyl)glucoside* | Flavonoids | 1.11E+07 | 9.00E+06 | 1.06E+07 | 2.55E+07 | 2.39E+07 | 1.79E+07 |
| pmb0608 | Chrysoeriol-7-O-(6''-malonyl)glucoside* | Flavonoids | 1.01E+05 | 1.09E+05 | 8.78E+04 | 2.04E+05 | 1.86E+05 | 2.62E+05 |
| ZBN0384 | Kaempferol glucosyl glucuronopyranosyl glucoside | Flavonoids | 6.79E+03 | 8.47E+03 | 1.14E+04 | 1.29E+04 | 2.28E+04 | 2.02E+04 |
| ZBN0303 | Kaempferol-3-O-(2''-O-acetyl)glucoside | Flavonoids | 7.24E+04 | 1.34E+05 | 1.13E+05 | 1.51E+05 | 1.79E+05 | 3.40E+05 |
| mws1454 | Persicoside | Flavonoids | 5.55E+04 | 2.39E+05 | 4.95E+04 | 2.24E+05 | 1.95E+05 | 2.88E+05 |
| Ladp004569 | portulacanone C malonyl glucoside | Flavonoids | 1.69E+07 | 9.08E+06 | 1.31E+07 | 2.29E+07 | 3.42E+07 | 2.29E+07 |
| Lmmn003398 | Kaempferol-3-O-(6''-O-acetyl)glucoside | Flavonoids | 1.14E+05 | 1.53E+05 | 9.71E+04 | 1.73E+05 | 3.17E+05 | 2.45E+05 |
| Zbsp004858 | Tricin-5-O-(6'-O-malonyl)glucoside | Flavonoids | 6.07E+05 | 8.50E+05 | 6.73E+05 | 2.20E+06 | 8.37E+05 | 1.25E+06 |
